# High-Entropy Sn_0.8_(Co_0.2_Mg_0.2_Mn_0.2_Ni_0.2_Zn_0.2_)_2.2_O_4_ Conversion-Alloying Anode Material for Li-Ion Cells: Altered Lithium Storage Mechanism, Activation of Mg, and Origins of the Improved Cycling Stability

**DOI:** 10.1021/acsami.2c11038

**Published:** 2022-09-12

**Authors:** Maciej Moździerz, Konrad Świerczek, Juliusz Dąbrowa, Marta Gajewska, Anna Hanc, Zhenhe Feng, Jakub Cieślak, Mariola Kądziołka-Gaweł, Justyna Płotek, Mateusz Marzec, Andrzej Kulka

**Affiliations:** † Faculty of Energy and Fuels, AGH University of Science and Technology, al. Mickiewicza 30, 30-059 Krakow, Poland; ‡ AGH Centre of Energy, AGH University of Science and Technology, ul. Czarnowiejska 36, 30-054 Krakow, Poland; § Faculty of Materials Science and Ceramics, AGH University of Science and Technology, al. Mickiewicza 30, 30-059 Krakow, Poland; ∥ Academic Centre for Materials and Nanotechnology, AGH University of Science and Technology, al. Mickiewicza 30, 30-059 Krakow, Poland; ⊥ State Key Laboratory of Space Power-Sources Technology, Shanghai Institute of Space Power-Sources, No. 2965 Dongchuan Road, Shanghai 200245, China; # Faculty of Physics and Applied Computer Science, AGH University of Science and Technology, al. Mickiewicza 30, 30-059 Krakow, Poland; ∇ Institute of Physics, University of Silesia, ul. 75 Pułku Piechoty 1, 41-500 Chorzow, Poland

**Keywords:** Li-ion cells, anodes, conversion and alloying reactions, high-entropy oxides, cycling stability, Li-storage mechanisms

## Abstract

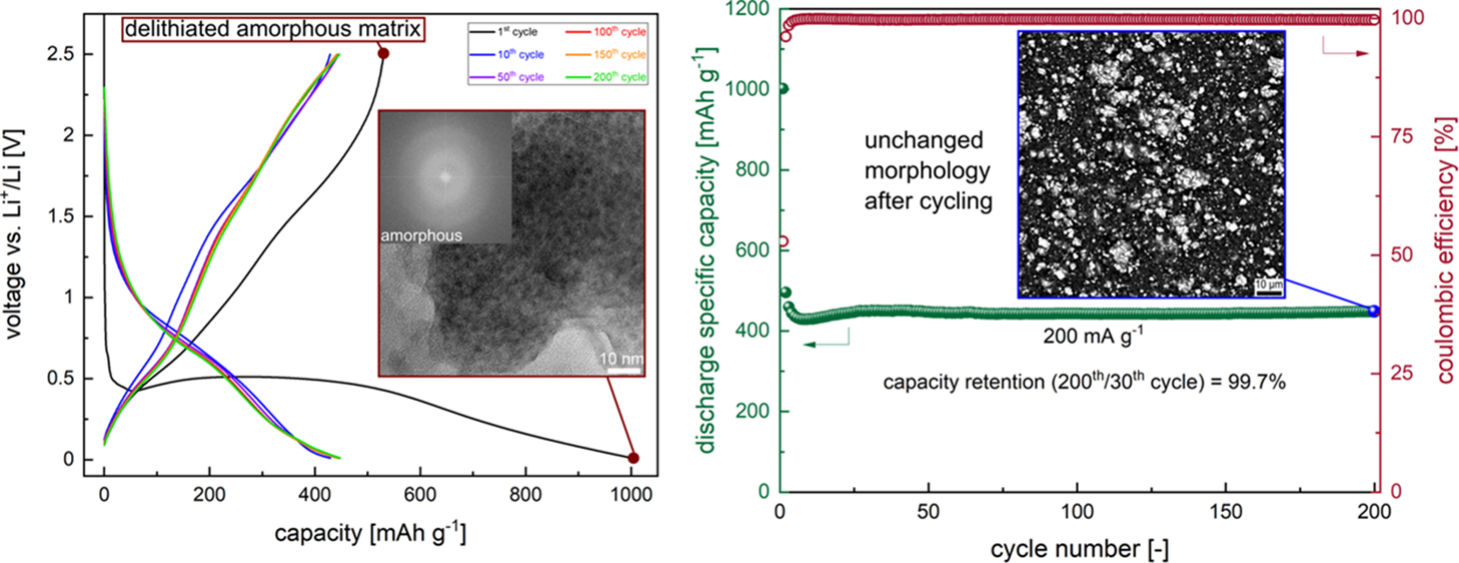

Benefits emerging from applying high-entropy ceramics in Li-ion technology are already well-documented in a growing number of papers. However, an intriguing question may be formulated: how can the multicomponent solid solution-type material ensure stable electrochemical performance? Utilizing an example of nonequimolar Sn-based Sn_0.8_(Co_0.2_Mg_0.2_Mn_0.2_Ni_0.2_Zn_0.2_)_2.2_O_4_ high-entropy spinel oxide, we provide a comprehensive model explaining the observed very good cyclability. The material exhibits a high specific capacity above 600 mAh g^–1^ under a specific current of 50 mA g^–1^ and excellent capacity retention near 100% after 500 cycles under 200 mA g^–1^. The stability originates from the conversion-alloying reversible reactivity of the amorphous matrix, which forms during the first lithiation from the initial high-entropy structure, and preserves the high level of cation disorder at the atomic scale. In the altered Li-storage mechanism in relation to the simple oxides, the unwanted aggregated metallic grains are not exsolved from the anode and therefore do not form highly lithiated phases characterized by large volumetric changes. Also, the electrochemical activity of Mg from the oxide matrix can be clearly observed. Because the studied compound was prepared by a conventional solid-state route, implementation of the presented approach is facile and appears usable for any oxide anode material containing a high-entropy mixture of elements.

## 1 Introduction

Nowadays, to construct fast-charging batteries with the desired high capacity and optimal operating voltage, as well as improved safety, the state-of-the-art intercalation-based graphite anode must be replaced by anodes with different chemistries operating based on new principles.^1−3^ Together with the implementation of new kinds of anodes, undoubtedly leading to substantial progress in the field of Li-ion batteries, many new challenging issues related to the different working mechanisms have emerged, making the actual commercialization of these materials limited almost only to composites with a small addition of Si, reversibly forming lithium intermetallics.^3−6^ In a search for a further improvement of the anodes’ electrochemical performance, it was proposed a few years ago to combine the alloying and conversion Li-storage mechanisms within a single compound (conversion-alloying materials, CAMs), benefiting from their advantages and confining the disadvantages^7,8^ (details of the idea of the CAM approach and its characteristics can be found in Supplementary Note 1). Despite overall improved electrochemical properties of CAMs, their poor cycling stability, mainly due to severe volumetric changes intrinsically related to both constituent types of reaction with Li, appears as the main limitation.^3,9,10^ As of today, the performance of CAM-based anodes in Li-ion cells is governed by the architecture of the particles and electrode, requiring rather expensive, sophisticated synthesis methods and/or carbonaceous additives.^7,8,11^

From another perspective, the most recent reports show that a novel group of materials, the so-called high-entropy oxides (HEOs), exhibits very promising electrochemical properties considering their use as cathodes^12−14^ and anodes^15−28^ in Li-ion cells. HEOs are regarded as multiprincipal component solid solutions characterized by high configurational entropy. The materials show significantly enhanced solubility limits and excellent structural stability, with most of the exceptional properties emerging from synergistic effects, beyond a simple rule of mixing.^12,29−32^ When the HEO-based anodes are compared, all have one thing in common: regardless of the synthesis method and resulting morphology, they always exhibit remarkable cycling stability, which distinguishes them from conventional analogues. The observed excellent cycling performance of HEOs is attributed, at least to some degree, to the influence of the high entropy on the phase stabilization.^15,21−24,26,27,33−36^ However, until now, it has not been properly addressed how exactly the entropy can stabilize phases in the ongoing electrochemical process, which leads to a decomposition of the initial structure. The current state of knowledge on the electrochemical working mechanisms of HEOs is summarized in Supplementary Note 2.

In our work, we have developed a novel concept of application of the high-entropy approach to CAMs, aiming for the creation of anode materials for Li-ion batteries characterized by excellent cycling stability, as well as good electrochemical performance, and most importantly, obtained using a simple synthesis method, without expensive additives. Utilizing an easily scalable solid-state route, we synthesized the spinel-structured Sn_0.8_(Co_0.2_Mg_0.2_Mn_0.2_Ni_0.2_Zn_0.2_)_2.2_O_4_ anode material, whose electrochemical properties significantly outperform conventional spinel-type CAMs. This allowed incorporating desired amounts of the respective elements into the anode material, being also homogenously distributed at the atomic level. We also explain the Li-storage mechanisms and origins of the reported very good cyclability, involving reversible lithiation of the amorphous matrix containing all the components well-mixed at the atomic scale, including electrochemically active Mg.

## 2 Experimental Section

### 2.1 Synthesis

The Sn-rich samples, Sn_0.8_(Co_0.2_Mg_0.2_Mn_0.2_Ni_0.2_Zn_0.2_)_2.2_O_4_ (further denoted as Sn0.8-ME5) and Sn(Co_0.2_Mg_0.2_Mn_0.2_Ni_0.2_Zn_0.2_)_2_O_4_ (further denoted as Sn1-ME5) HEOs, as well as the SnZn_2_O_4_ reference material, were synthesized using a typical solid-state method. The following reactants were weighed in the selected stoichiometric proportions: SnO_2_ (Alfa Aesar, 99.9%), Co_3_O_4_ (Alfa Aesar, 99.9985%), MgO (Alfa Aesar, 99.95%), MnO (Alfa Aesar, 99.99%), NiO (Alfa Aesar, 99.998%), and ZnO (Alfa Aesar, 99.99%). The powders were mixed in isopropyl alcohol for 20 min in a high-energy ball mill Spex SamplePrep 8000 M using zirconia balls and subsequently dried. The ball-to-powder weight ratio was ca. 3:1. The obtained precursors were formed into 10 mm diameter pellets using a uniaxial hydraulic press under a pressure of 250 MPa. The green bodies were then free-sintered at 1200 °C for 20 h followed by cooling down to room temperature with a furnace. The as-obtained sinters were ground with an agate mortar into powders. In some cases, there was an additional step including another pelletizing and sintering under the same conditions.

### 2.2 Characterization (XRD, SEM/EDS, DLS, TEM/HR-TEM/STEM/EDS/SAED, Raman Spectroscopy, Mössbauer Spectroscopy, and XPS)

The as-obtained ground powders were characterized by the X-ray diffraction (XRD) method conducted in θ–θ Bragg–Brentano geometry using a Panalytical Empyrean diffractometer with CuKα radiation equipped with a PIXcel3D detector within the 10–110° range at room temperature. The typical measurement lasted for 51 min with a resolution of 0.013°. Panalytical HighScore software (ICDD PDF4+ 2021 database) was used for qualitative phase analysis. Quantitative analysis of XRD data was performed via Rietveld refinements using GSAS-II.^37^ Morphology and chemical composition of the oxides were investigated using scanning electron microscopy (SEM) in secondary electrons (SEs) and/or backscattered electrons (BSEs) combined with energy-dispersive X-ray spectroscopy (EDS) analysis using a ThermoFisher Scientific Phenom XL Desktop SEM equipped with a silicon drift detector. The applied accelerating voltage was 15 kV. Raman spectroscopy studies were performed at room temperature on a Thermo Scientific DXR3 Raman Microscope using a 532 nm green laser, 1800 grooves/mm grating, and long working distance optical objective (50×), in the measurement range 50–1800 cm^–1^ with a resolution of 0.5 cm^–1^. The estimated spot size was equal to 1.1 μm. The ^119^Sn Mössbauer spectra were collected in the transmission geometry at room temperature by using a ^119^Sn source in a Ca^119m^SnO_3_ matrix. The 23.875 keV γ-rays were detected using a proportional counter LND 45431. A palladium foil of 0.05 mm thickness was used to reduce the tin K X-rays concurrently emitted by this source. The velocity scale was calibrated by taking the spectrum of α-Fe. This calibration method is a standard one because of the narrowest and most well-defined spectral lines of the ^57^Fe isotope among all the known Mössbauer isotopes. Transmission electron microscopy (TEM) measurements (including high-resolution TEM (HR-TEM), scanning TEM using high-angle annular dark field detector (STEM HAADF), EDS, and selected area electron diffraction (SAED)) were performed using an FEI Tecnai TF20 X-TWIN (FEG) microscope (Thermo Fisher Scientific) equipped with an EDS detector, operating at 200 kV accelerating voltage. Samples for the TEM investigations were prepared by drop-casting (materials suspended in isopropyl alcohol) on carbon-coated copper TEM grids. The X-ray photoelectron spectroscopy (XPS) analyses were carried out in a PHI VersaProbeII Scanning XPS system using monochromatic Al Kα (1486.6 eV) X-rays focused to a 100 μm spot and scanned over the area of 400 μm × 400 μm. The photoelectron take-off angle was 45°, and the pass energy in the analyzer was set to 46.95 eV to obtain high-energy resolution spectra for the O 1s, Ni 2p, Co 2p, Mn 2p, Zn 2p, and Sn 3d_5/2_ regions. A dual beam charge compensation with 7 eV Ar^+^ ions and 1 eV electrons was used to maintain a constant sample surface potential regardless of the sample conductivity. All XPS spectra were charge-referenced to the unfunctionalized, saturated carbon (C–C) C 1s peak at 285.0 eV. The operating pressure in the analytical chamber was less than 5 × 10^–9^ mbar. Deconvolution of spectra was carried out using PHI MultiPak software. Spectrum background was subtracted using the Shirley method. The information depth was estimated to about 5 nm within the geometry of the spectrometer. Soft X-ray absorption spectroscopy (XAS) measurements were conducted in the 400–1400 eV range, probing Mg K-edge and Mn, Co, Ni, Zn L_3_-, and L_2_-edges. Data were collected in partial fluorescence yield (PFY) mode, providing bulk information up to ca. 100 nm, at room temperature under high vacuum (≤10^–8^ mbar). Experiments were performed at the end station of XAS beamline at the National Synchrotron Radiation Centre SOLARIS (Krakow, Poland). The particle size distribution was measured through the dynamic light scattering (DLS) technique using Malvern Mastersizer 3000 apparatus in deionized water as the dispersant.

### 2.3 Electrochemical Studies

For the electrode layer preparation, powders of Sn0.8-ME5, Sn1-ME5, and reference SnZn_2_O_4_ were used, after grinding them for 30 min in a mortar. In a typical process, an electrode was prepared by mixing the active material, Timcal Graphite & Carbon Super P (MTI Corporation), and poly(vinylidene fluoride) binder (PVDF, HSV900 Arkema) in 70:20:10 weight ratio in *N*-methyl pyrrolidone (NMP, Alfa Aesar, 99.5%) using a high-speed homogenizer Polytron PT 2500 E to obtain a slurry. The obtained mixture was coated on a Cu foil (12 μm thick, MTI Corporation) via the doctor blade method and then dried. The mass loading of the active material was in a range of 1.5–2.0 mg cm^–2^. Subsequently, CR2032 coin half-cells with Li foil (0.75 mm thick, Alfa Aesar, 99.9%) as the counter electrode, glass microfiber (Whatman) and polymer Celgard separators, and commercial electrolyte (1 M LiPF_6_ in 1:1 (v/v) ethylene carbonate (EC): diethyl carbonate (DEC), Sigma Aldrich) were assembled inside an Ar-filled glovebox (UNILab MBraun, Ar, H_2_O < 0.1 ppm, O_2_ < 0.1 ppm). The obtained batteries were analyzed electrochemically through the galvanostatic charge/discharge (GDC), electrochemical impedance spectroscopy (EIS), and cyclic voltammetry (CV) techniques on a Biologic VMP3 electrochemical workstation. All the measurements were conducted at 23 °C in a thermostat. The specific capacities and currents were calculated taking into account the mass of the active material.

For the preparation of optimized electrodes, the same Sn0.8-ME5 powder (manually ground for 30 min) was mixed with Timcal Graphite & Carbon Super P (MTI Corporation), carboxymethyl cellulose (CMC, MTI Corporation), and styrene-butadiene rubber (SBR, 48 wt % water solution, MTI Corporation) in 70:20:5:5 or 65:25:5:5 ratio in deionized water overnight. The mass loading of the active material was controlled in a range of 1.2–1.5 mg cm^–2^. The rest of the procedure was similar to that for the PVDF binder, except for the electrolyte: in this case, 1 M LiPF_6_ in 1:1 (v/v) EC:DEC with 5 wt % addition of fluoroethylene carbonate (FEC, Sigma-Aldrich, 99%) and 1 wt % of vinylene carbonate (VC, Alfa Aesar, 97 + %) was used.

### 2.4 Ex Situ Investigations

The Sn0.8-ME5 oxide was investigated ex situ from half-cells using a series of techniques: TEM, SEM, EDS, Mössbauer spectroscopy, and XAS. The experimental conditions for those techniques were the same as described in Section 2.2. All the batteries with the studied oxide were slowly (dis-)charged to the selected electrochemical state (specific current of 20 mA g^–1^), relaxed for 48 h (except for ex situ XAS after 20 cycles, where the applied current was 100 mA g^–1^, and the long-term ex situ SEM studies for which the charge/discharge conditions are described in the text), and disassembled inside the Ar-filled glovebox. In the case of TEM, SEM, and XAS measurements, to ensure optimal experimental conditions, the electrodes after disassembling were soaked in DEC electrolyte solvent. For TEM studies, ex situ samples were ultrasonicated in isopropyl alcohol and prepared by drop-casting on carbon-coated copper TEM grids. In the case of Mössbauer spectroscopy, samples together with a Pb mask were sealed with Kapton tape and embedded in epoxy resin. All the samples were transferred to the respective apparatus in sealed bags under an Ar atmosphere to prevent oxidation.

### 2.5 Operando XRD and EIS Measurements

For the operando XRD measurements, the self-supported electrode layers were prepared by mixing Sn0.8-ME5 powder, Timcal Graphite & Carbon Super P, and PVDF (70:10:20 ratio) in acetone as a solvent. The amount of binder within the self-standing electrode layer was increased compared with a typical electrode on a Cu foil to avoid cracking during cell’s assembly and battery work. The slurry was spread onto the glass support via the doctor blade method and dried in air. Subsequently electrodes of 8 mm diameter were punched out. The loading of the active material was in a range of ca. 7 mg cm^–2^. The prepared electrodes were assembled with a Li counter electrode, a glass microfiber separator (Whatman), and a commercial electrolyte (1 M LiPF_6_ in 1:1 (v/v) EC:DEC), in a custom-made electrochemical cell with a beryllium window, compatible with a Panalytical Empyrean diffractometer (the same as described in Section 2.2.). The experiment was conducted during the first discharge/charge cycle using a one-channel Biologic potentiostat/galvanostat with a specific current of 30 mA g^–1^ during discharge and 10 mA g^–1^ during charge in the 0.01–2.5 V potential range. The investigated 2θ range was 10–65°, with 45 min per single measurement (0.013° resolution).

Electrochemical impedance measurements as a function of (de-)lithiation state (here referred to as operando EIS) were conducted for a typical half-cell with an Sn0.8-ME5 active material, a PVDF binder, and a commercial electrolyte. EIS measurement was conducted every 150 mAh g^–1^ during the first discharge/charge cycle (including measurement of the fresh cell) under the constant specific current equal to 30 mA g^–1^ in the voltage range of 0.01–2.5 V, resulting in eight spectra for discharge and four spectra for charge. Before each of the spectra was collected, the cell had been relaxed for 3 h. EIS measurement was performed in the frequency range of 10^–1^ to 10^6^ Hz with 10 mV disturbance amplitude. EIS was also performed for a reference symmetrical Li–Li cell. All of the spectra were analyzed with the use of the distribution of relaxation times (DRT) approach, conducted using free software DRT tools^38^ with the regularization parameter equal to 10^–3^. For Li-ion batteries, relaxation time corresponds to a time constant of a given process occurring in a cell. Thanks to DRT analysis, it is possible to resolve processes characterized by similar time constants, which usually cannot be done by typical fitting of EIS data using equivalent circuit models. Derivation and precise description of the DRT approach can be found in ref (38). Prior to the DRT calculations, all the spectra had been validated in terms of Kramers–Krönig relations.

## 3 Results

### 3.1 Structure of the New Sn_0.8_(Co_0.2_Mg_0.2_Mn_0.2_Ni_0.2_Zn_0.2_)_2.2_O_4_ HEO Spinel

Numerous inverse 4-2 spinels with a general formula [*A*^2+^]^tetrahedral^[*A*^2+^*B*^4+^]^octahedral^O_4_ are known, in which +2 cations from the 3d metals group (as well as Mg^2+^) are present in both the tetrahedral and the octahedral sites.^7,8^ Because the B^4+^ cation can be Sn^4+^, this naturally gives rise to a group of spinel-type CAMs, in which a favorable ratio between tin, working on a basis of the alloying reaction with Li, and selected 3d metal A^2+^ cations showing (typically) the conversion-type reactivity, is present. The B^4+^:A^2+^ ratio, equal to 1:2, seems vital for the reported very good electrochemical performance of such CAMs.^7,8,39^ Bearing in mind that A^2+^ can be selected among many ions, this allows for an easy implementation of the high-entropy approach. More details about the selection of the discussed system and the reactivity of chosen elements with Li can be found in Supplementary Note 3.

**Figure 1:**
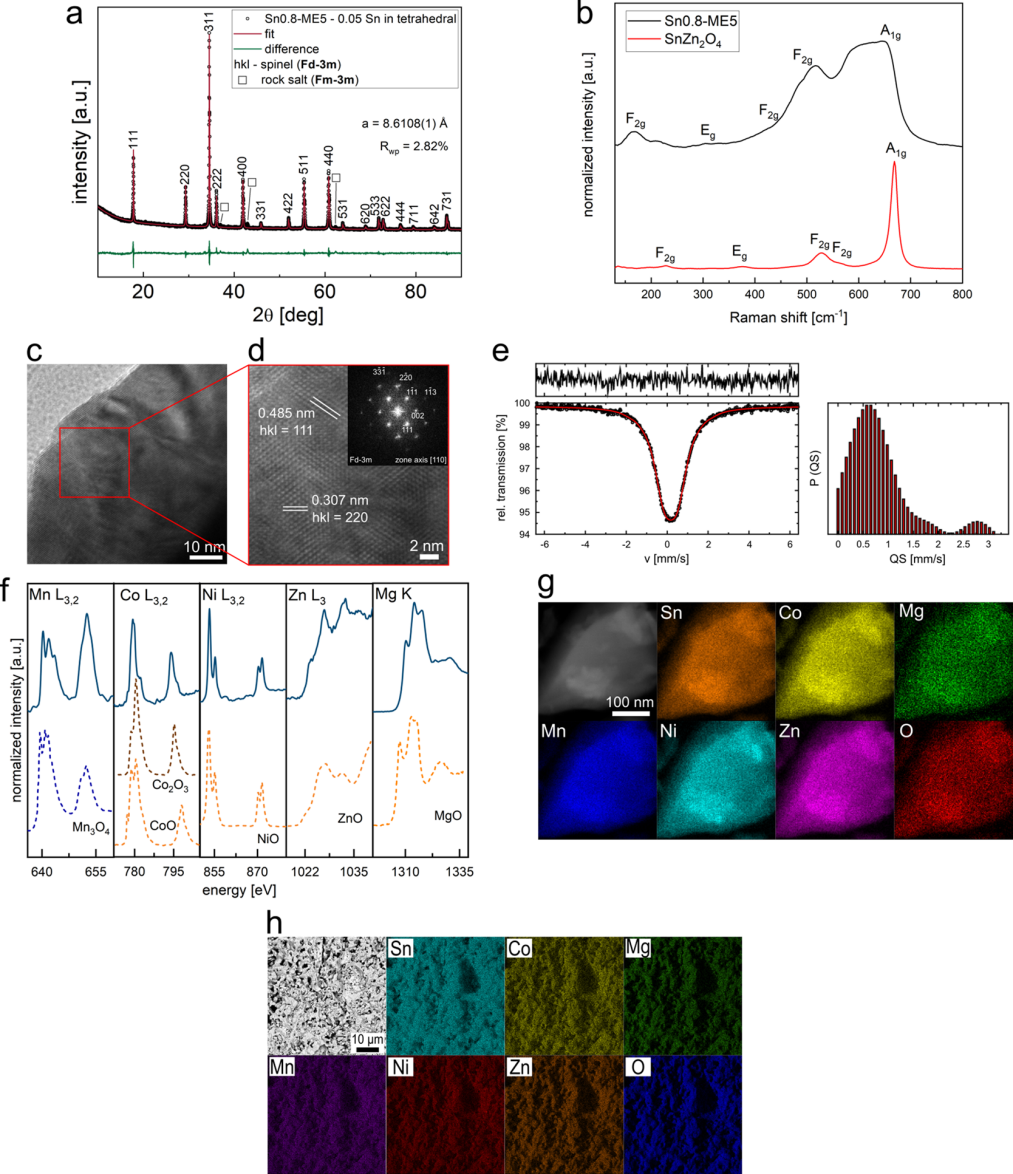
Structural studies of Sn0.8-ME5: (a) XRD data with Rietveld refinement assuming partial (0.05) transfer of Sn to tetrahedral positions, *R*_wp_ = 2.82%, for all Sn in octahedral sites *R*_wp_ = 2.96% (not shown); minor amount of the rock salt-structured second phase is marked with black squares; Miller indices correspond to the spinel phase (*Fd*-3*m*), *a* is the lattice parameter, and *R*_wp_ is the weighted profile *R*-factor of the refinement. (b) Raman spectrum for Sn0.8-ME5 (mean from 10 measurements at different positions on the sample’s surface) compared with a measured spectrum for conventional SnZn_2_O_4_; vibrational modes assigned based on ref (41). (c,d) HR-TEM image measured for manually ground powder (c), and the zoomed region (d) with the inset showing the corresponding FTT pattern with indexed spots assigned to the spinel structure, [110] zone axis. (e) Room-temperature ^119^Sn Mössbauer spectrum fitted assuming one quadrupole doublet with the calculated parameters presented in Table S2 and difference spectrum at the top, together with the quadrupole splitting distribution curve. (f) XANES spectra for Mg K-edge and Mn, Co, Ni, Zn L_3_-, and L_2_-edges measured in PFY mode with reference spectra for simple oxides from the literature.^46−50^ (g) STEM image with the corresponding EDS map showing the uniform distribution of elements, measured for manually ground powder. (h) SEM image with the corresponding EDS map measured on a gently polished pellet’s cross-section.

Initially, we attempted to maintain the stoichiometric spinel composition and synthesize Sn(Co_0.2_Mg_0.2_Mn_0.2_Ni_0.2_Zn_0.2_)_2_O_4_ HEO (further denoted as Sn1-ME5), using a conventional solid-state route. However, the XRD data showed that while the sample contained the majority of the desired *Fd*-3*m* spinel phase, the precipitated SnO_2_ phase could be clearly observed. To eliminate this unwanted phase, we synthesized a composition with the tin deficiency (in relation to the conventional 4-2 Sn-based spinels), Sn_0.8_(Co_0.2_Mg_0.2_Mn_0.2_Ni_0.2_Zn_0.2_)_2.2_O_4_ (further denoted as Sn0.8-ME5). The XRD pattern with performed Rietveld refinement for the material (Figure 1a) shows that we successfully obtained the spinel-structured HEO, with only a negligible amount of the rock salt-structured secondary phase (3.3 wt %). A more detailed description of the phase composition of Sn0.8-ME5 and Sn1-ME5 is provided in Supplementary Note 4, Figure S1, and Table S1. Interestingly, a better Rietveld fit could be obtained assuming a small amount of tin present also in tetrahedral positions, indicating an increasing cation disorder and a partial transformation toward random spinel.^40^

Comprehensive TEM, Raman, and ^119^Sn Mössbauer spectroscopy studies allowed describing the structural features of the considered Sn0.8-ME5 HEO. The bands visible in the Raman spectrum correspond to the spinel-type structure, with five active modes typical of the *Fd*-3*m* space group^40−42^ (Figure 1b). However, large broadening as well as the emergence of additional peaks suggests a high level of cation disorder and structural distortion (Supplementary Note 5). The presence of the spinel-type structure is further proven by the HR-TEM, with all the observed spots in the Fast Fourier Transform pattern assigned to the *Fd*-3*m* space group (Figures 1c,d and S2). The observed character of the Mössbauer spectrum can be explained with two possible approaches (Figures 1e, S3a, Table S2, and Supplementary Note 5). First, similarly to the Raman spectrum, it hints at a significant crystal lattice distortion, which is considered typical of the high-entropy materials, because of the various ionic radii of cations present in the structure (the so-called lattice distortion effect).^28,43^ Another possible explanation is in line with the Rietveld refinement fit, suggesting the location of tin in two different crystallographic positions: mainly in the octahedral sites, but with a small amount also in tetrahedral sites. Based on the fitted spectrum, it can be concluded that all Sn exhibits +4 oxidation state,^40^ as it is expected for the spinel-type CAMs. This is also consistent with XPS measurements (Figure S3b and Supplementary Note 5). According to the XPS data, the oxidation states of all other cations in the material are +2, except for Mn^3+^. Also, based on the fitted O 1s spectrum, it can be stated that the content of oxygen vacancies (at least on the surface) is rather negligible, contrary to some of the other high-entropy spinels.^34,44,45^ Additionally, we conducted synchrotron-based XAS measurements. X-ray absorption near-edge structure (XANES) spectra for the Mg K-edge and Mn, Co, Ni, Zn L_3_-, and L_2_-edges collected in PFY mode are presented in Figure 1f, together with the reference spectra for single oxides. For Mn, the spectrum characterized with two features at 639 eV (L_3_-edge) and 650 eV (L_2_-edge) is in good agreement with reference Mn_3_O_4_ one,^46^ indicating that the Mn oxidation state is mixed +2/+3. For Co, features at 778 and 793 eV correspond to Co L_3_- and L_2_- edges. The first feature is split into two smaller peaks (779.3 and 779.9 eV), which is in good agreement with the CoO reference.^47^ However, the presence of an asymmetrical shoulder toward higher energies most probably indicates the existence of a mixed valency +2/+3, but with a predominant amount of Co^2 + .^^47^ The L_3_- and L_2_- edges of Ni for the pristine sample occur at energy 872.7 and 869.8 eV. Both peaks are split into two smaller ones, indicating the presence of Ni^2+^.^48^ The measured L_3_ edge of Zn corresponds well to the referenced ZnO.^49^ In the case of the Mg K-edge, the spectrum is similar to that of the referenced MgO,^50^ indicating +2 oxidation state. The differences between Co and Mn oxidation states from XAS results compared with XPS data (where only Co^2+^ and Mn^3+^ were detected) likely originate from the tendency of Mn to oxidize on the sample’s surface (XAS measured in PFY mode provides bulk information), and minor content of Co^3+^ in the sample (better sensitivity of XAS). Nevertheless, based on the results from both methods, it can be stated that manganese is the cation responsible for charge compensation resulting from the tin deficiency. The observed tendency of Mn to exhibit a higher oxidation state can be correlated with precipitation of SnO_2_ in the Sn1-ME5 sample and a lack of such impurity in the Sn0.8-ME5 material. The performed EDS chemical analysis (Figure S4, Table S3, and Supplementary Note 6) indicates that the obtained composition of the Sn0.8-ME5 is close to the nominal. The homogeneous distribution of the elements in the material was confirmed through the EDS mappings performed at the nanoscale (STEM, Figure 1g) and the microscale (SEM, Figure 1h). To summarize, our results unambiguously show that the nonequimolar spinel-structured HEO with a high amount of Sn (compared to the content of other cations) and a significant cation mixing can be obtained using a facile, easily scalable solid-state route.

### 3.2 Electrochemistry

**Figure 2:**
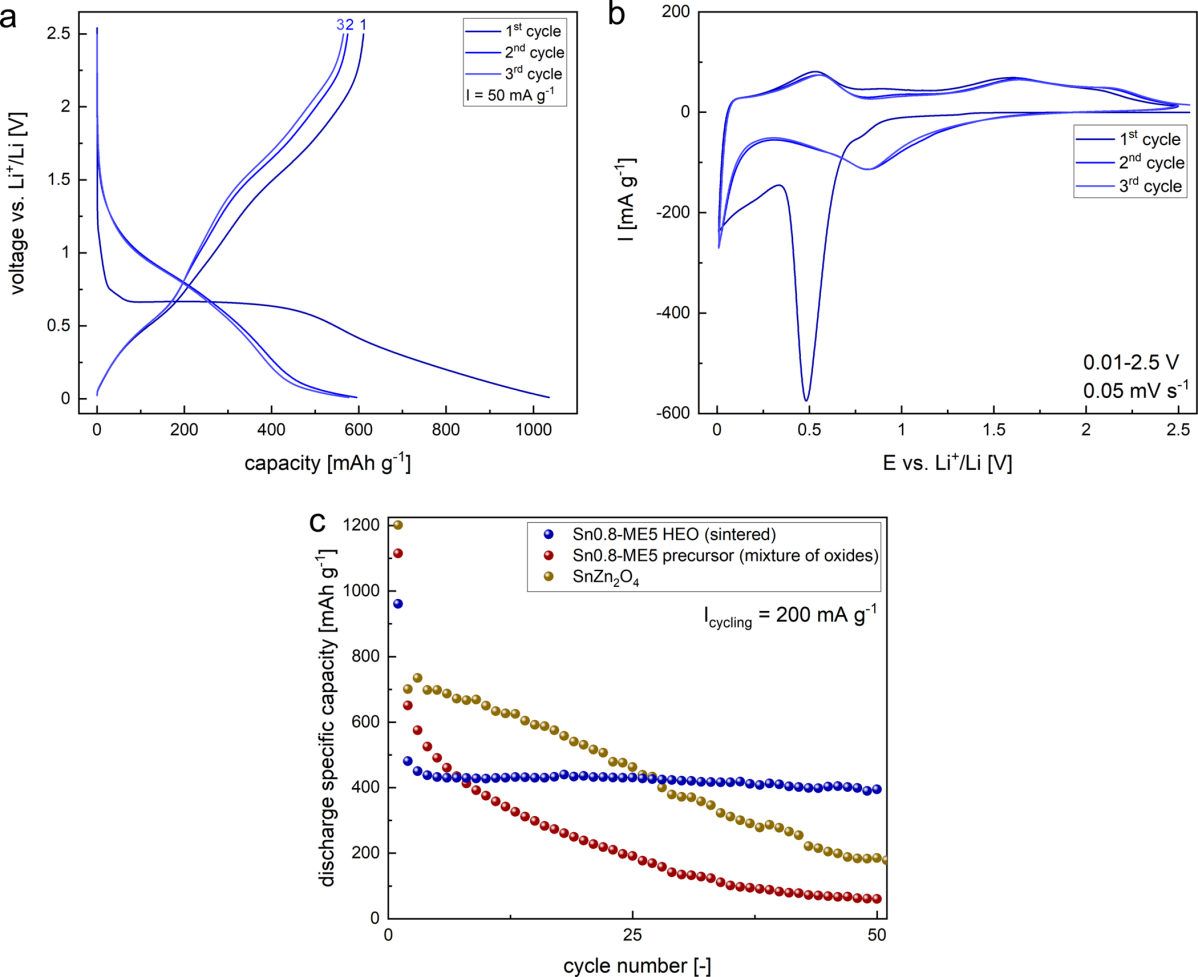
Electrochemical properties of Sn0.8-ME5 HEO: (a) GDC curves for the electrode with the PVDF binder in the voltage range of 0.01–2.5 V under a specific current of 50 mA g^–1^ for three initial cycles; numbers on top indicate the cycle number; (b) CV curves for three initial cycles in the voltage range of 0.01–2.5 V with a scan rate of 0.05 mV s^–1^. (c) Comparison of cycling performance for the Sn0.8-ME5 HEO, the precursor (not a sintered mixture of single oxides with the same chemical composition as HEO), and conventional spinel-structured SnZn_2_O_4_ CAM obtained via the solid-state method at a specific current of 200 mA g^–1^ for 200 cycles in the voltage range of 0.01–2.5 V.

For typical spinel-type CAM electrode materials, the initial structure is fully converted after the first lithiation and not restored during the subsequent cycles.^7,8^ To investigate the influence of the high-entropy spinel structure of the Sn0.8-ME5 oxide on the electrochemical properties, the material was tested in half-cells using the GDC method (Figure 2a) and compared with a pristine mixture of respective oxides (precursor after ball milling, Figure S5a). While a high initial loss of lithium, typical of conversion and alloying anodes,^7,8^ can be observed for both electrodes (initial coulombic efficiency: ICE_HEO_ = 59%, ICE_precursor_ = 63%), the character of the curves is markedly different. Multiple voltage plateaus are visible for the precursor, originating from the electrochemical reactions of each of the constituent oxides. The curves are much more smooth for the HEO, indicating a changed lithiation mechanism. The differences between electrodes are even more pronounced in the CV curves, with the three initial cycles presented in Figure 2b for the HEO and Figure S5b for the mixture of oxides. In the case of the precursor, as can be expected for the multiphase electrode, there are numerous peaks, also present in the subsequent scans. There is a clear drop in the peak current values between cycles, suggesting poor reversibility. Regarding the HEO, during the first lithiation, the main peak centered at ca. 0.5 V is visible, corresponding to the decomposition of the spinel structure and the formation of the solid-electrolyte interphase (SEI) film. The relatively lower formation voltage compared to the precursor indicates a more kinetically stable SEI.^51^ The increase of a specific current at voltages below ca. 0.45 V can be related to the ongoing alloying reaction.^9^ Subsequent anodic and cathodic scans are well overlapped, indicating good reversibility. The observed CV peaks can be generally divided into two main redox pairs, with peaks centered at ca. 0.02/0.55 V (lower potentials, suggesting (de-)alloying reaction^9^) and ca. 0.82/1.63 V (higher potentials, suggesting conversion reaction^10^). It should be underlined that the recorded CV peaks are very broad, suggesting that both reactions are occurring gradually in a wide potential range. Such a phenomenon is an effect of the excellent mixing of active elements at the atomic scale,^52^ originating from the initial random distribution of cations in the spinel phase, which is apparently also maintained during cycling. Taking into consideration that the precursor is a multiphase material with the majority of phases other than spinel (hence the effective charge transfer may be different in relation to the spinel-type HEO), it is worth comparing the electrochemical performance of the proposed HEO conversion-alloying anode material with other conventional materials from the group of CAMs. The cycling performance at a specific current of 200 mA g^–1^ for Sn0.8-ME5 HEO, the precursor, and spinel-structured SnZn_2_O_4_ CAM (prepared according to the same methodology) is presented in Figure 2c. The poor cyclability observed for SnZn_2_O_4_ is in line with the previous studies.^53^ Interestingly, the initial lithiation/delithiation capacities are higher for both the precursor and SnZn_2_O_4_, equal to 1268/761 and 1202/693 mAh g^–1^, respectively, while for the sintered HEO the values are 1047/632 mAh g^–1^. However, the superior stability of the Sn0.8-ME5 electrode is evident, as it delivers the highest capacity of all the studied materials after the 28^th^ cycle. The results indicate a lower overall lithiation level of the Sn0.8-ME5 active material and further emphasize that the electrochemical reaction mechanism is significantly altered for the HEO.

**Figure 3:**
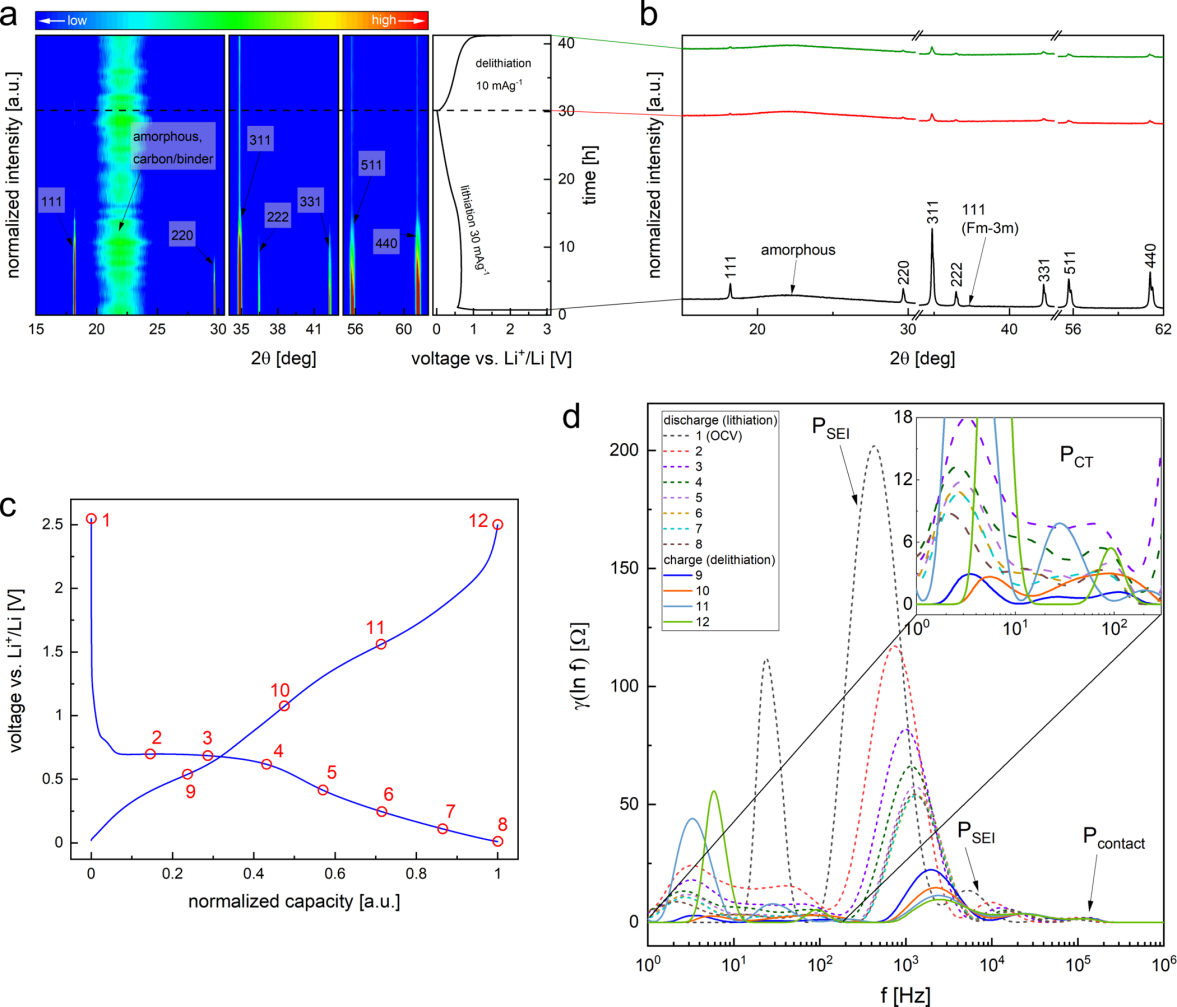
Operando analysis of Sn0.8-ME5: (a) XRD contour plot together with corresponding voltage characteristics in the 0.01–2.5 V range under a discharge current of 30 mA g^–1^ and a charge current of 10 mA g^–1^; Miller indices correspond to the *Fd*-3*m* spinel phase. (b) XRD patterns measured for pristine (black), fully lithiated (red), and fully delithiated (green) electrodes; Miller indices correspond to the spinel phase, with a minor peak from a rock salt-structured secondary phase, which is visible only for the pristine electrode and disappears upon electrochemical reactions. (c) Normalized first cycle GDC curves for the electrode with the PVDF binder measured in the 0.01–2.5 V voltage range under a specific current of 20 mA g^–1^; marked points correspond to EIS measurements at different (de-)lithiation states. (d) Distribution function of relaxation times as a function of frequency presented in the selected frequency range for the electrode at different (de-)lithiation states with the number corresponding to the GDC characteristics in (c); inset shows the zoomed region for the peaks related to the CT polarization; P_CT_—peaks related to the CT polarization; P_SEI_—peaks corresponding to the SEI-related and Li counter electrode polarization; P_contact_—peaks from the contact impedance.

The altered electrochemical behavior of the Sn0.8-ME5-based electrode, compared with the studied precursor, as well as with the conventional CAMs (without electrocatalytic additives like graphene oxide, please see refs.^7,8,53−56^ and our experimental data for SnZn_2_O_4_), requires a detailed explanation. For this purpose, we have employed operando XRD (Figure 3a,b) and EIS (Figure 3c,d) methods. We found that the spinel structure is progressively decomposing with the lithiation (decrease of the intensity of XRD peaks). The most prominent structural changes occur at around 50% of the first discharge normalized capacity (between points 4 and 5 in Figure 3c), indicating that the long plateau corresponds to the spinel decomposition and formation of the amorphous and/or nanocrystalline structure. No further changes were observed in the XRD patterns, meaning that the well-crystallized phases are not rebuilt at any stage of (de-)lithiation. Reasons for the incomplete conversion in this experiment (low-intensity *Fd*-3*m* phase peaks remain after full lithiation) are explained in Figure S6 and Supplementary Note 7. More information about properties of the electrode can be extracted from operando EIS measurements analyzed using the distribution of relaxation times (DRT) technique.^38^ The results are presented in Figure 3d, where the integral area of each peak is connected with the polarization resistance of a given process.^57^ The raw EIS data as well as the assignment of the observed peaks to the various polarization effects, together with their interpretation, are discussed in Figure S7 and Supplementary Note 8, while the DRT methodology is described in Section 2.5. Here, we focus on the qualitative change of peaks assigned to the charge transfer polarization (P_CT_), mainly related to the properties of studied active material.^57,58^ In the conversion reaction process, the charge transfer (CT) resistance is gradually decreasing (points 1–8 on the normalized curve in Figure 3c), which hints conversion of constituents of the HEO to a metallic state. The most significant modification in the P_CT_ region occurs between points 1 and 2, indicating a substantial change of the physicochemical properties of the Sn0.8-ME5,^58^ even at the beginning of lithiation (connected with the decomposition of the spinel observed via operando XRD measurement). Also, P_CT_ is slightly shifted toward lower frequencies, indicating slower kinetics of CT upon lithiation. During the initial stages of delithiation, P_CT_ is centered at higher frequencies, and the resistance further drops in the low-voltage range (points 9 and 10), suggesting that dealloying of the metals improves the transport properties. Then, the CT resistance significantly increases between points 10 and 11, reaching the highest value at the end of charge (point 12). It can be explained by the oxidation of species at higher potentials vs. Li^+^/Li, as oxides are generally poor conductors compared with metals. Consequently, DRT results support the electrochemical character of the Sn0.8-ME5 electrode suggested by the GDC and CV measurements.

**Figure 4:**
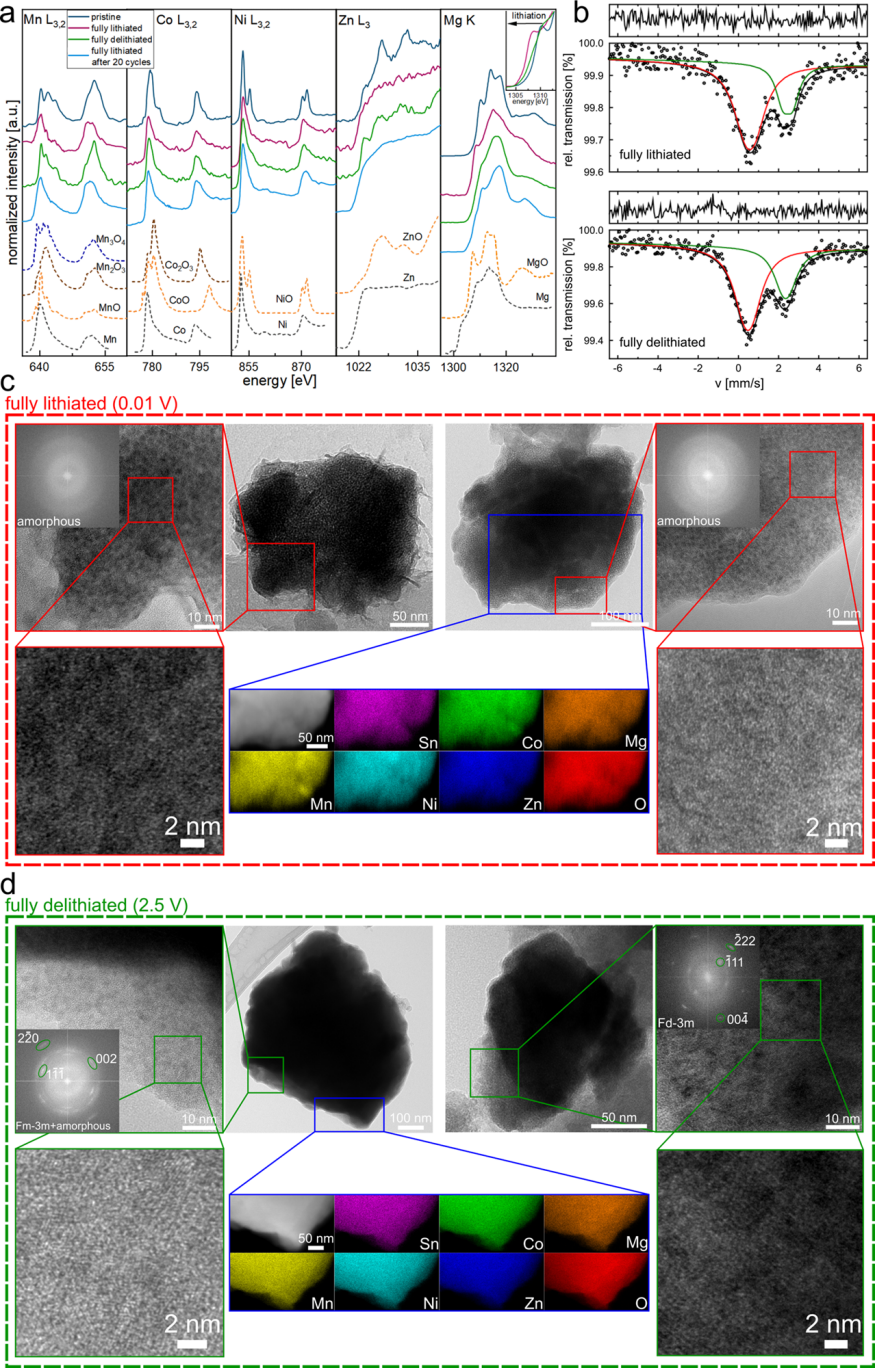
Ex situ studies of Sn0.8-ME5: (a) XANES spectra the Mg K-edge and Mn, Co, Ni, Zn L_3_-, and L_2_-edges measured in PFY mode for pristine, fully delithiated, and fully lithiated after 1^st^ and 20^th^ cycles electrodes, with reference spectra for simple oxides from the literature.^46−50,60,61^ (b) Room-temperature ^119^Sn Mössbauer spectra fitted assuming two quadrupole doublets with the calculated parameters presented in Table S4 and difference spectrum at the top for fully lithiated and delithiated samples. (c) Ex situ TEM analysis for the fully lithiated electrode: bright-field TEM images with corresponding HR-TEM analysis and FTT patterns of the whole image, showing amorphous character of the sample (left-hand side) and a mixture of amorphous phase with rock salt-like features (right-hand side, zoomed HR-TEM image); Also, the STEM/EDS map is presented, confirming maintained homogeneity of elemental distribution of the lithiated electrode. (d) Ex situ TEM analysis for the fully delithiated electrode: bright-field TEM images with corresponding HR-TEM analysis and FTT patterns of the whole image, showing a mixture of rock salt (*Fm*-3*m*) and amorphous phases (left-hand side) and spinel-structured (*Fd*-3*m*) nanocrystallites (right-hand side). The STEM/EDS map shows that the homogeneity is still preserved upon delithiation.

To get a deeper insight into the electrochemical mechanisms, we performed ex situ measurements at different stages of (de-)lithiation using XAS (Figure 4a), Mössbauer spectroscopy (Figure 4b), and TEM/STEM/EDS (Figure 4c,d). The detailed methodology behind the interpretation of XAS and Mössbauer spectra is described in Supplementary Note 9, while here we present only the derived conclusions. To recall, spectroscopic data indicate that Sn is at +4 state, and all the other elements of the pristine HEO material are at +2 oxidation state, except for Co^2+/3+^ and Mn^2+/3+^. Of importance, for the fully lithiated state, surprisingly, only ca. 29% of Sn is reduced to the metallic state, while the rest remains at +4, contrary to the conventional tin-containing oxide anodes, where typically all the Sn is reduced.^7,8,59^ Also, the values of hyperfine parameters for Sn^0^, derived from Mössbauer spectra fit (Table S4 and Supplementary Note 9), suggest that highly lithiated Li–Sn intermetallics are not formed.^59^ Regarding Co and Ni ions, they are almost entirely reduced in the conversion reaction, with only a weak signal suggesting residue of higher oxidation states. The same can be stated for Mn; however, it becomes fully activated after 20 cycles. Zn is also found in reduced form at the end of the discharge. Unexpectedly, we found that typically inactive Mg^2+^^15^ also takes part in the initial conversion reaction of the HEO, eventually giving a mixture of Mg^0/2+^, with a predominant amount of the metallic state. While this phenomenon was previously inferred for (Co_0.2_Cu_0.2_Mg_0.2_Ni_0.2_Zn_0.2_)O HEO,^21^ here it is directly evidenced for the first time. The XAS spectra measured at the intermediate lithiation states during the first cycle (Figure S8 and Supplementary Note 9) clearly show that the reduction of all the ions occurs progressively, except for Mg, which is not reduced until the end of the discharge. Therefore, in order to activate Mg^2+^, other ions must first form metallic particles, which then catalyze the reaction of Mg, similarly as proposed for (Co_0.2_Cu_0.2_Mg_0.2_Ni_0.2_Zn_0.2_)O.^21^

As directly evidenced by the ex situ TEM studies (Figures 4c and S9a), the active material undergoes almost full amorphization upon lithiation. We detected only a minor amount of crystallites in the lithiated state, which was identified as a spinel-structured phase (Figure S9b) with some trace of a rock salt-type phase (Figure 4c right-hand side, zoomed HR-TEM image, Figure S9a,c,d). For the latter one, the measured d-spacings match well the MgO phase (*Fm*-3*m* space group; the observation is in line with the partial presence of Mg^2+^ visible in the XAS spectrum for the fully lithiated electrode). Also, a trace signal of Li_2_O, which is expected to be a mostly amorphous product of the conversion reaction, can be found in the SAED pattern (Figure S9d). Moreover, the electrode was found to be highly homogeneous, within the spatial resolution of STEM/EDS mapping.

For the fully delithiated material, the proportion between Sn^0/4+^ is the same as for the lithiated one (ca. 1:2), meaning that Sn participates only in the (de-)alloying process. Zn and Mn were found to work reversibly, returning to their original oxidation states. Interestingly, Co and Ni exhibit only a slight tendency for oxidation upon delithiation, yielding a limited reversible activity (likewise, this was observed for the (Co_0.2_Cu_0.2_Mg_0.2_Ni_0.2_Zn_0.2_)O anode^21^). Partially, Mg is oxidized back to +2 state, which is also clearly visible through TEM imaging (Figure 4d left-hand side, zoomed HR-TEM image, Figure S10a,b), showing exsolution of the rock salt-type MgO from the amorphous matrix. The origin of the existence of Mg^0^ in the fully delithiated electrode is not understood yet, and therefore, it requires further studies. After 20 cycles at the fully lithiated state, there is even more Mg^2+^ than Mg^0^, as compared to the fully delithiated electrode after the first cycle, leaving inactive MgO particles. However, the clearly changing proportion between 0 and + 2 states during battery work indicates that this redox pair is electrochemically active. Similarly as for the lithiated material, we found nanocrystallites of a spinel-structured oxide in the delithiated material, being the electrochemically inactive part of the electrode (Figure 4d right-hand side, Figure S10c). The rest of the active material remains amorphous. Notably, the segregation of elements upon delithiation was not detected (STEM/EDS maps), meaning that nanocrystalline MgO is distributed homogeneously within the electrode.

The presented above results indicate the presence of numerous unique features related to the reversible (de-)lithiation of the Sn0.8-ME5 HEO material. In the next section, we present a model explaining this behavior, as well as an outlook for the high-entropy anode materials in the Li-ion technology.

## 4 Discussion and Outlook

The complex electrochemical working mechanism of the Sn-rich Sn0.8-ME5 HEO anode can be described as follows. After the first lithiation, the formed homogeneous and amorphous multicomponent matrix can be reversibly (de-)lithiated at lower potentials (alloying-type reaction) and partially, but also reversibly, oxidized at higher potentials (conversion-like reaction). Both reactions contribute to lithium storage. Because the reversible electrochemical activity of the amorphous matrix has never been observed in conventional CAMs,^7,8^ we believe it is the most important finding reported here, as the amorphization is known to be beneficial regarding cyclability of the anode materials.^62,63^ Consequently, highly lithiated crystalline intermetallics (e.g., from the Li–Sn system), characterized by significant volume changes on cycling,^64^ are not formed. We postulate that this change of the electrochemical reaction mechanism is due to the presence of well-mixed cations, originating from the initial structure introduced by the high-entropy approach. The role of the high entropy is therefore rather limited to ensuring the atomic-scale mixing of the elements. At the same time, it is generally considered that an electrochemically driven solid-state amorphization can be caused by the accumulation of dislocations. They can be formed upon lithiation because of the emerging electrochemical stresses, and this gives the necessary energy surplus to go far from equilibrium and create the amorphous matrix.^65^ This accumulation can be significantly facilitated in the high-entropy materials.^66−69^ It appears similar to the solid-solution strengthening effect, due to the high disorder and significant lattice distortion, both of which could be detected for the Sn0.8-ME5 HEO. On the other hand, the once formed matrix remains amorphous upon delithiation, as there are no (clearly defined) grain boundaries,^70^ where the crystallization occurs effectively. Moreover, working against the demixing of the disordered matrix characterized by a high structural distortion and stresses is energetically unfavorable. The crystallization can be also suppressed by kinetical limitations and the energy barriers associated with the strongly varied nearest neighbor surrounding of the cations. As evidenced in this work, there is only a gradual bulk (de-)lithiation of the multicomponent amorphous matrix ongoing. This explains why no separate electrochemical reactions are observed at potentials characteristic of each individual element of the HEO. Additionally, a minority of the inactive nanocrystalline phases is also exsolved from the amorphous matrix (rock salt and spinel). Those phases additionally buffer the volume changes.^26,36^ All the discussed atomic-scale effects, while resulting in the somewhat lowered capacity due to the lower level of lithiation, contribute greatly to the enhanced cycling stability. Consequently, the whole electrochemical behavior can be described in detail, without referring to the undefined “high-entropy stabilization effects.” This answers the question from the abstract about how the multicomponent solid-solution material enables reaching excellent long-term stability during cycling.

**Figure 5:**
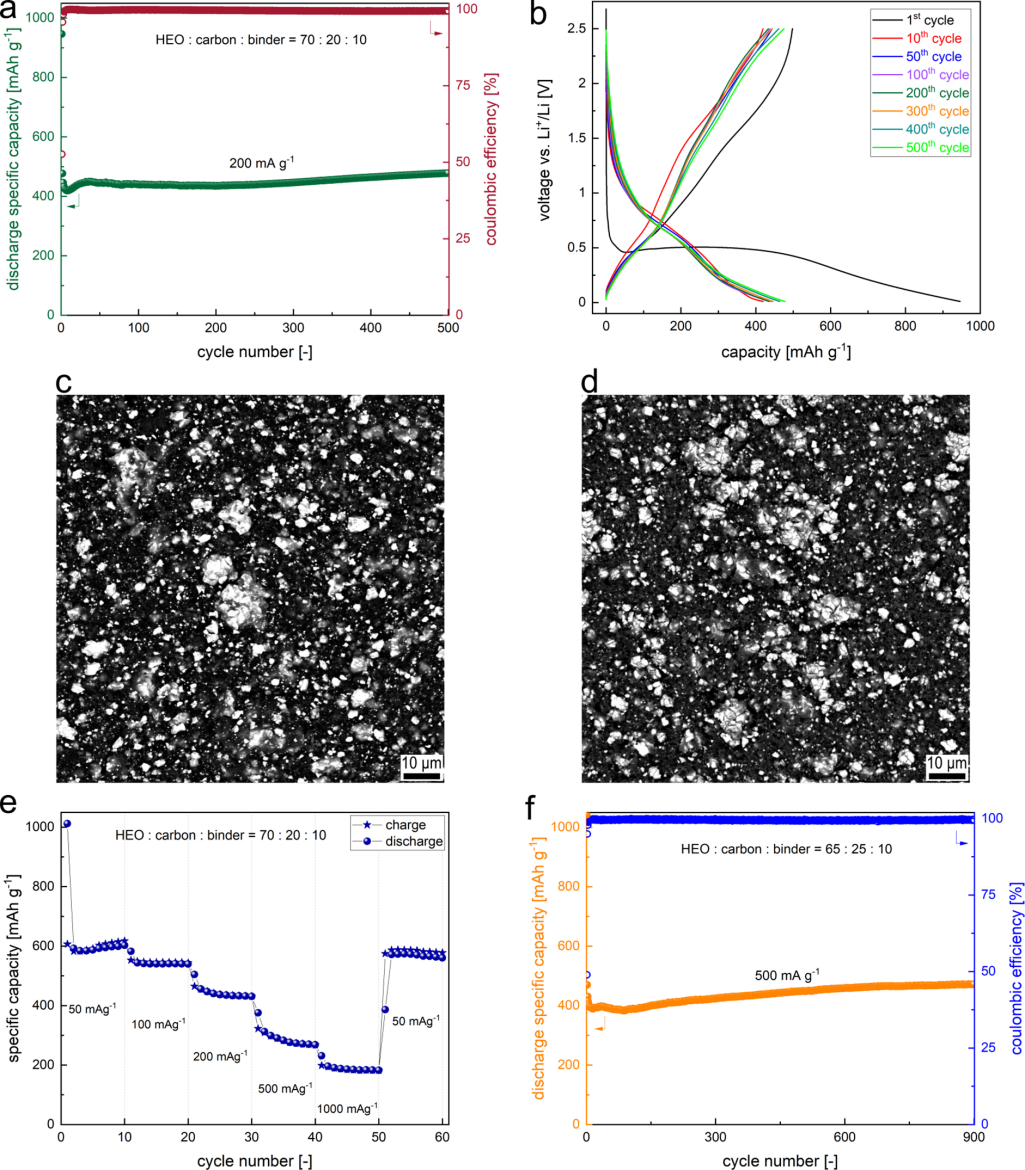
Cycling stability of the optimized Sn0.8-ME5-based electrode; The binder is CMC/SBR, and the electrolyte is 1 M LiPF_6_ in 1:1 (v/v) EC:DEC with 5 wt % FEC and 1 wt % VC addition. (a) Capacity retention under a specific current of 200 mA g^–1^ in the voltage range of 0.01–2.5 V for 500 cycles for the 70:20:10 electrode’s composition. (b) Corresponding GDC curves for 1^st^, 10^th^, 50^th^, 100^th^, 200^th^, 300^th^, 400^th^, and 500^th^ cycles. (c,d) SEM micrographs in the BSE mode for the pristine electrode (c) and the fully lithiated electrode (d) after 200 cycles at a specific current of 200 mA g^–1^. Higher magnification micrographs and micrographs in the SE mode can be found in Figure S11. (e) Rate capability test in the voltage range of 0.01–2.5 V for the 70:20:10 electrode’s composition with the specific current values given on the graph. (f) Long-term cycling under a specific current of 500 mA g^–1^ in the voltage range of 0.01–2.5 V for 900 cycles for the 65:25:10 electrode’s composition. Increased carbon additive content was used because it is beneficial for cycling under high currents.^71^

As proof of concept, we have optimized our electrode in terms of the type of binder and electrolyte additives. As visible in Figure 5a, the solid-state synthesized Sn0.8-ME5 micrograined active material (average primary grain size equal to ca. 2 μm, Figure S4c and Supplementary Note 6) delivers a high average reversible capacity of 450 mAh g^–1^ under 200 mA g^–1^ specific current. The GDC curves between 50 and 500 cycles are well overlapped, indicating excellent reversibility (Figure 5b). Up to 200 cycles, the average discharge capacity drop per cycle is equal only to 0.24 mAh g^–1^ (excluding the first cycle), with an excellent capacity retention of 99.7% (comparing 200^th^ and 30^th^ cycles). Moreover, as visible in the ex situ SEM studies for the electrode after 200 cycles (Figures 5c,d and S11), there are virtually no morphological changes, microcracks, and elemental segregation visible after cycling. Importantly, the observed grains of the active HEO material are amorphous, as there is no signal from large crystalline phases detected through other techniques (except for the nanosized regions). Above around 200 cycles, the capacity starts to slightly increase with further cycling. This effect is typical of anodes with large particles involving the conversion-type reaction and is likely related to activation processes.^15,24^ It may also be caused by the “quasi-reversible” formation of the SEI with the continuous electrolyte decomposition, as the pronounced capacity increase occurs mainly in very low and very high potential regions^49^ (Figure 5b). This phenomenon has been previously observed for other spinel-type CAMs as well.^49^ Considering the capacity retention of the Sn0.8-ME5-based electrode in the full cycled range (comparing 500^th^ and 2^nd^ cycles), it reaches a value as high as 100%. The material also exhibits great stability in the rate capability test (Figure 5e), with almost fully recovered capacity after returning to the lowest current (capacity retention equal to 97%, comparing 52^nd^ and 2^nd^ cycles). It is worth mentioning that performance under higher currents of the studied electrode can be further improved by changing the ratio of active material to carbon additive^71^ to binder from 70:20:10 to 65:25:10. The long-term stability test for 900 cycles at a specific current of 500 mA g^–1^ is presented in Figure 5f, with the corresponding GDC curves shown in Figure S12a. While similarly as for the 70:20:10 electrode the increase of capacity for prolonged cycling can be observed, this result further proves the great cyclability of the developed HEO, even for higher rates. The influence of the optimized carbon content on the accessible reversible capacity at a specific current of 200 mA g^–1^ for 200 cycles is presented in Figures S12b,c. In the case of the carbon content-optimized electrode, the observed average discharge capacities in the entire cycled range are outstanding and equal to 569 mAh g^–1^ for 200 mA g^–1^ and 438 mAh g^–1^ for 500 mA g^–1^. Additionally, to investigate the performance at even higher currents, for the cell cycled initially 750 times at 500 mA g^–1^, additional tests were conducted, comprising 50 cycles at 1000 mA g^–1^, followed by 50 cycles at 2000 mA g^–1^. The obtained, stabilized capacities were ca. 280 and 165 mAh g^–1^, respectively. In general, to our knowledge, this is so far the best ever observed cyclability for the solid-state synthesized CAM,^8,53^ proving that the high-entropy approach can be successfully applied for preparing anodes using facile and low-cost synthesis routes, instead of going toward sophisticated and complex synthesis methods.^72^

**Figure 6:**
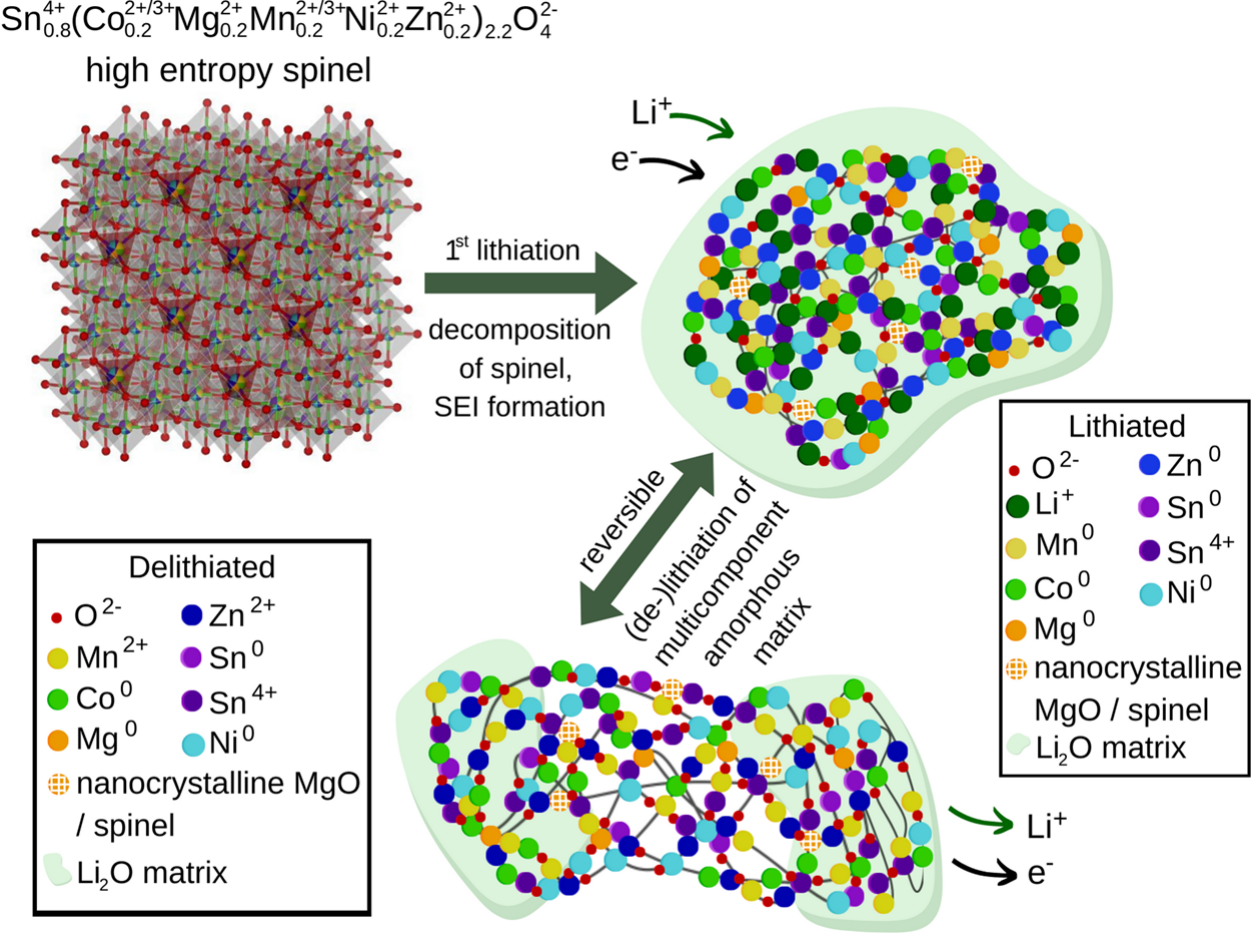
Schematic of the proposed Li-storage mechanism for the Sn0.8-ME5 conversion-alloying anode material. The initially disordered and distorted high-entropy spinel lattice decomposes during the first lithiation with a concurrent SEI film formation. In this process, the lithiated homogeneous multicomponent amorphous matrix, containing Sn^0/4+^, O^2–^, Co^0^, Mg^0^, Mn^0^, Ni^0^, and Zn^0^, is created. Mg^2+^ is partially reduced to Mg^0^. There is a small amount of residual electrochemically inactive nanocrystalline phases: MgO (rock salt) and spinel. Amorphous-like Li_2_O is formed as well. The fully lithiated matrix contains a significant amount of Sn in the oxidized (+4) state, together with some amount of oxygen ions. The main amorphous matrix is reversibly (de-)lithiated through both the alloying-like reaction (low potentials) and conversion-like reaction (higher potentials). Only Zn and Mn undergo fully reversible conversion (between 0 and + 2 states), delivering ca. 30% of the capacity. The remaining 70% of the capacity is ascribed to the alloying-like reaction of the amorphous matrix. Upon delithiation, the recrystallization does not occur, as working against demixing of the disordered matrix is energetically unfavorable, and there are no grain boundaries for a facile crystallization.

The proposed Li-storage mechanism for Sn0.8-ME5 HEO is schematically summarized in Figure 6. Additionally, the capacity for the first lithiation and the reversible capacity were compared with the calculated theoretical capacities (taking into consideration the experimental capacity from the rate capability test under a low specific current of 50 mA g^–1^, Supplementary Note 10). As discussed above, only Zn and Mn ions, embedded in the amorphous matrix, can undergo fully reversible conversion reactions. This corresponds to ca. 30% of the reversible capacity. The remaining 70% of the capacity must originate from the reversible alloying-like reaction of the amorphous matrix, that is occurring without a change of the oxidation state of any particular element, but rather through electron exchange within the entire homogeneous matrix. Also, the observed capacity is significantly lower when compared with the theoretical capacity of the HEO system, considering all the possible conventional reactions (Supplementary Note 10). This underlines the fact that the Li-storage mechanism is indeed significantly changed for the Sn0.8-ME5, as well as that the excellent long-term stability of the HEO related to the altered (de-)lithiation mechanism results in lowered capacity.

Based on our results, guidelines for the future development of HEO-based anodes can be provided. Further studies of the Sn-rich conversion-alloying HEOs should focus on the optimization of the chemical composition, for which the high-entropy approach provides vast possibilities. In particular, this optimization should aim to improve performance by increasing the amount of the alloying-based elements per mole of HEO (nonequimolar approach), which also seems to be a good direction for the development of the conventional CAMs.^39^ In the light of our results, HEOs do not necessarily need to be equimolar to achieve excellent cycling stability in Li-ion cells, as long as multiple main elements, characterized by various physicochemical properties, are well-mixed within the initial structure. Therefore, we believe that the maximization of the configurational entropy is in fact not crucial here.

In summary, the presented approach of designing a high-entropy conversion-alloying anode material shall contribute to the improvement of the performance of Li-ion batteries. It allows achieving excellent long-term stability of the anode material manufactured using a simple solid-state reaction route. It also provides great opportunities for further optimization, for example, by increasing the amount of the elements undergoing alloying reaction, which shall lead to the increased energy density of the cells (because of the higher capacity and lower operation voltage of the anode material).

## Supporting Information Available

Literature description about CAMs and HEOs; description of the selection of the system; XRD analysis with XRD patterns for Sn1-ME5 and Sn0.8-ME5 compositions and electrochemical performance of Sn1-ME5; TEM and SAED analysis of the pristine Sn0.8-ME5; Raman, Mössbauer, and XPS analysis together with corresponding spectra for pristine Sn0.8-ME5; studies of morphology and chemical composition of Sn0.8-ME5 powder via SEM, TEM, EDS, and DLS methods; electrochemical properties of the precursor for HEO synthesis; ex situ XRD patterns compared with operando XRD measurement; operando EIS with DRT analysis and presentation of the raw EIS spectra; analysis of the ex situ Mössbauer spectroscopy data; analysis of the ex situ XAS data with measured spectra for partially lithiated Sn0.8-ME5-based electrodes; complementary ex situ TEM results for both lithiated and delithiated Sn0.8-ME5-based electrodes; ex situ SEM analysis results together with EDS maps for the optimized electrode (pristine and after 200 cycles); cycling performance for the Sn0.8-ME5-based electrode with different active material to carbon ratios; and calculation of the theoretical capacity of the Sn0.8-ME5 anode material (PDF)
