# Supplementary material for: High-Entropy Sn0.8(Co0.2Mg0.2Mn0.2Ni0.2Zn0.2)2.2O4 Conversion-Alloying Anode Material for Li-Ion Cells: Altered Lithium Storage Mechanism, Activation of Mg, and Origins of the Improved Cycling Stability
Source: ACS Appl Mater Interfaces. 2022 Sep 12;14(37):42057–70. doi: 10.1021/acsami.2c11038 (PMC9501916; doi:10.1021/acsami.2c11038)
Supplement: Supplementary file 1 — am2c11038_si_001.pdf [file am2c11038_si_001.pdf]

Supporting Information

High entropy $Sn_{0.8}(Co_{0.2}Mg_{0.2}Mn_{0.2}Ni_{0.2}Zn_{0.2})_{2.2}O_4$ conversion-alloying anode material for Li-ion cells: altered lithium storage mechanism, activation of Mg, and origins of the improved cycling stability

Maciej Moździerz[1], Konrad Świerczek[1,2,*], Juliusz Dąbrowa[3], Marta Gajewska[4], Anna Hanc[1], Zhenhe Feng[5], Jakub Cieślak[6], Mariola Kądziołka-Gaweł[7], Justyna Płotek[1], Mateusz Marzec[4], Andrzej Kulka[1]

[1]AGH University of Science and Technology, Faculty of Energy and Fuels,
al. Mickiewicza 30, 30-059 Krakow, Poland
[2]AGH Centre of Energy, AGH University of Science and Technology,
ul. Czarnowiejska 36, 30-054 Krakow, Poland
[3]AGH University of Science and Technology, Faculty of Materials Science and Ceramics,
al. Mickiewicza 30, 30-059 Krakow, Poland
[4]Academic Centre for Materials and Nanotechnology, AGH University of Science and Technology,
al. Mickiewicza 30, 30-059 Krakow, Poland
[5]State Key Laboratory of Space Power-Sources Technology, Shanghai Institute of Space Power-Sources, no. 2965 Dongchuan Road, Shanghai 200245, China
[6]AGH University of Science and Technology, Faculty of Physics and Applied Computer Science,
al. Mickiewicza 30, 30-059 Krakow, Poland
[7]University of Silesia, Institute of Physics,
ul. 75 Pułku Piechoty 1, 41-500 Chorzow, Poland
[*]xi@agh.edu.pl

Supplementary Note 1: Features of the conversion-alloying anode materials

Alloying/dealloying reaction

Regarding candidate alloying-type anodes for Li-ion batteries, such as Si, Zn, Sn, their main advantages are related to the very high theoretical gravimetric/volumetric capacities (e.g. 3578 mAh g^{-1} and 2194 mAh cm^{-3}, considering $Li_{15}Si_4$ as the final product of Si alloying[1], 410 mAh g^{-1} and 1476 mAh cm^{-3}, considering LiZn as the final product of Zn alloying[2], 993 mAh g^{-1} and 2111 mAh cm^{-3}, considering $Li_{4.4}Sn$ as the final product of Sn alloying[3]), reasonably low working potentials (e.g. ca. 0.40 V for Si, 0.16 V for Zn, and 0.38 V for Sn, all vs. Li^+/Li), which enables to achieve high energy density, as well as low voltage hysteresis[4–6]. These features make alloying-based materials attractive substitutes for the state-of-the-art graphite anode. However, the main problems preventing their commercialization application are low stability and significant capacity fading during cycling, caused by enormous volume changes upon lithiation/delithiation (even up to 420% for silicon), aggregation of particles, and high initial irreversible loss of lithium[4,7]. Currently, only a small amount of Si addition to the graphite anode could be successfully applied (< 10 wt%) in the commercial cells, which is introduced between larger graphite particles[8].

Conversion reaction

The conversion materials, which typically are transition metal (TM) oxides, also exhibit high theoretical capacities (e.g. 890 mAh g^{-1} for Co_3O_4 [9], 937 mAh g^{-1} for Mn_3O_4 [10], 718 mAh g^{-1} for NiO [11]), yet lower than in the case of the alloying-based anodes[5]. However, it should be stressed that while such materials also suffer from severe capacity fade upon cycling, the volume changes for the conversion reaction are in the range of 40-100%, which is substantially lower than for metallic anodes[4]. Nevertheless, if the materials operating on the conversion principle shall become a commercial reality, it is necessary to decrease their relatively high operating voltage (e.g. average voltage of 2.0 V for Co_3O_4, 1.2 V for Mn_3O_4, 1.9 V for NiO), and mitigate significant voltage hysteresis caused by different reaction pathways of lithiation and delithiation[4]. Substantial irreversible consumption of lithium in the first cycle is also profound in this case[4,5,12].

Combination of both mechanisms - conversion-alloying materials (CAMs)

Considering CAM approach (e.g. $SnCo_2O_4$, $Sn_{0.9}Fe_{0.1}O_2$ [4]), in comparison to the alloying reaction, such a combination of operating principles allows for buffering of the volume changes by the emerging Li_2O matrix (formed upon conversion), as well as for preventing particle aggregation with the simultaneous formation of the conductive nano-network of TM metallic particles. On the other hand, compared to pure conversion materials, thanks to the contribution of the alloying mechanism, almost all of the crucial electrochemical parameters can be improved: the overall operating voltage is lowered, the capacity is increased, and the voltage hysteresis is decreased. Due to the synergistic effects, the electrochemical properties of CAMs are not a simple weighted average of the properties of the elements contributing to each of the mechanisms, but a significant

enhancement of performance can be achieved. However, capacity fading during cycling still remains a problem, as it is caused by substantial volume variations and structural changes characteristic for both involved mechanisms involved[4,5,12].

Supplementary Note 2: Literature reports on working mechanisms of HEOs

Reports on the electrochemical working mechanisms of HEO-based Li-ion anodes are still quite limited, and can be divided into two main groups depending on the structure of the active material: rock salt-type oxides, e.g. $(Co_{0.2}Cu_{0.2}Mg_{0.2}Ni_{0.2}Zn_{0.2})O$ [13], and spinel-type materials, e.g. $(Co_{0.2}Cr_{0.2}Fe_{0.2}Mn_{0.2}Ni_{0.2})_3O_4$ [14].

The first report on the application of HEOs as an anode in Li-ion cells was referred to rock salt-type $(Co_{0.2}Cu_{0.2}Mg_{0.2}Ni_{0.2}Zn_{0.2})O$ [13] oxide. The five-component material was compared in terms of cycling stability with its quaternary subsystems (e.g. $(Co_{0.25}Cu_{0.25}Mg_{0.25}Ni_{0.25})O$), demonstrating the superiority of the high entropy approach. The behavior upon (de-)lithiation was summarized as the conversion into metallic nanoparticles and Li_2O responsible for Li storage, together with the preservation of an inert entropy-stabilized rock salt matrix. This mechanism appears to be markedly different from the conventional conversion-based anodes. The authors highlighted the crucial role of the inactive (in the applied potential range) Mg^{2+}, which helps to maintain a stable structure during battery operation. In the follow-up papers regarding this HEO[15,16], excellent cyclability was also observed, which was once again ascribed to the presence of the entropy-stabilized matrix. Moreover, in those articles, the influence of the inactive Mg was further studied, proving its significant contribution to the structural stabilization during (de-)lithiation. Recently, an alternative working mechanism of $(Co_{0.2}Cu_{0.2}Mg_{0.2}Ni_{0.2}Zn_{0.2})O$ anode material has been discussed[17]. In this case, it was shown that products of the initial conversion reaction are amorphous. Thus, the (de-)lithiation phenomena were studied through the suitable X-ray absorption spectroscopy method. The authors proposed a complex two-stage process. The first part involves the irreversible and incomplete conversion of Co, Cu, and Ni, giving a mixture of further inactive metallic particles and corresponding oxides, yielding excellent cycling stability. In the second stage, metallic Mg and Zn, also formed upon conversion, undergo the reversible alloying reaction with Li, which is responsible for the recorded high capacity. Interestingly, in contrast to the previous studies of the rock salt-structured HEO, here Mg was found to be electrochemically active, but the provided proof was indirect. Still, the authors emphasized the role of the stabilizing effect of the configurational entropy, as the initial structure was preserved for 60% of charge capacity during the first cycle (but not in the whole potential range).

Excellent stability during charge/discharge cycles was also reported for all spinel-structured HEOs investigated so far[14,18–23]. Noteworthy, there is a much larger variety of spinel-type HEO compositions compared with only one type of rock salt-structured HEO (and its derivatives, e.g. Li-doped[24] or with various Mg contents[16]). Regarding the working mechanisms of the high entropy spinels, the first study concerned $(Co_{0.2}Cr_{0.2}Fe_{0.2}Mn_{0.2}Ni_{0.2})_3O_4$ material, storing Li through

conversion reaction[14]. Ex-situ HR-TEM and XRD measurements indicated that an amorphous HEO, without any component segregation, was formed for both the fully lithiated and delithiated electrode. This observation was employed to explain the great stability of capacity after numerous cycles - the amorphization should suppress separation of the elements, and buffer volume changes during cycling. Furthermore, the multiplicity of cations in the equimolar HEO, resulting in a high configurational entropy, was suggested to provide many active sites for Li storage, as well as to contribute to the great long-term performance. Similar conclusions and explanation of the excellent electrochemical properties have been drawn for $(Co_{0.14}Cr_{0.14}Fe_{0.14}Mn_{0.14}Ni_{0.14}Li_{0.14}X_{0.14})_3O_4$ (X = Cu/Mg/Zn) equimolar high entropy anode materials[18]. Additionally, the enhanced performance was associated with formation of the oxygen vacancies due to the presence of Li^+ in the pristine samples. From structural studies of $(Al_{0.2}CoCrFeMnNi)_{0.58}O_{4-\delta}$ HEO after cycling in Li-ion cell, it was suggested that the recorded exceptional cycling stability originates from the entropy-stabilized crystal structure acting as a matrix (formed upon incomplete conversion reaction), which blocks volume changes[19]. In another work[20], a novel $(Cu_{0.2}Fe_{0.2}Mg_{0.2}Ti_{0.2}Zn_{0.2})_3O_4$ HEO was also shown to exhibit remarkable stability during cycling even under high loads (e.g. 2 Ag^{-1}), which was explained in terms of unique properties of HEOs, resulting in robust structural stability, and enhanced electronic conductivity. Noteworthy, in addition to Li-storage via the conversion reaction, (de-)alloying of Zn was also observed at low potentials vs. Li^+/Li for this material. The lithium storage mechanisms have also been studied for $(Co_{0.2}Fe_{0.2}Mn_{0.2}Ni_{0.2}Ti_{0.2})_3O_4$ oxide[21]. The key role of the inactive Ti, forming nanocrystalline $LiTi_2O_4$ phase during the initial lithiation, was emphasized, while the rest of the cations was shown to undergo reversible conversion reaction. Interestingly, it was inferred that after full delithiation the initial high entropy spinel structure is fully recovered. Although the pristine material was micrometer-sized, according to operando transmission X-ray microscopy, significant volume changes during battery work did not occur. This phenomenon was attributed to the presence of a buffering matrix, which also prevents agglomeration of the metal nanoparticles, formed thanks to “the high entropy stabilization of the lattice”. Most recently, non-equimolar high entropy spinel oxide (with a general formula of $(Co_{0.29}Cr_{0.12}Fe_{0.08}Mn_{0.29}Ni_{0.21})_3O_4$, according to the studies of chemical composition), has been investigated as the conversion-based anode material, with the focus on the excellent long-term stability of capacity, especially when compared with the traditional conversion-type oxides[22]. Using the ex-situ XRD method combined with TEM studies, the authors showed that the crystalline oxide framework is retained during (de-)lithiation, similarly to other types of spinel HEOs. It was proposed that in this case the effect is caused by a strong entropy-induced phase stabilization. Most recently, the same non-equimolar HEO was studied once again, this time using a combination of TEM and electron energy-loss spectroscopy, in order to get a deeper insight into the Li-storage mechanisms on the atomic level[23]. The authors observed clear crystallinity at nanoscale at different stages of (de-)lithiation, as well as prominent separation of elements. The identified phases, both characterized by the spinel structure, namely $Mn_xCr_yFe_{3-x-y}O_4$ and $LiNi_xCo_{1-x}O_2$, were found to be stable in the entire investigated potential range. The presence of this kind of framework, being in agreement with the previous reports on this composition[22], was found to be responsible for the excellent cycling performance. Metallic nanoparticles, created in the process of full lithiation of

the electrode, likely diffuse back to the spinel structure during delithiation through the growth of residual spinel oxide grains (acting as crystallization nuclei). The reason given for the conversion reaction stop at the detected spinel phases (instead of going toward full decomposition of the initial structure) involved the so-called “high entropy stabilization effect of HEOs”. Based on the literature data discussed above, it is clear that the working principle of HEO-based anodes, as well as the origins of their great cyclability, are still controversial and not fully understood. In particular, despite different structural changes upon (de-)lithiation, the high entropy stabilization effect is always mentioned to explain the enhanced cyclability.

Supplementary Note 3: Selection of the system and reactivity of different elements with Li

Selection of the Sn-Co-Mg-Mn-Ni-Zn-O system and the targeted spinel-type structure for the synthesis of novel HEOs was based on the literature data for conventional CAMs, known for their good electrochemical performance when used as anodes in Li-ion cells: $SnZn_2O_4$ [25,26], $SnCo_2O_4$ [27,28], $SnMg_2O_4$ [29,30], $SnMn_2O_4$ [31], and $SnNi_2O_4$ [32] (all are fully inversed spinels with general formula $[A^{2+}]^{tetrahedral}[A^{2+}B^{4+}]^{octahedral}O_4$ [5,33]). From a point of view of electrochemistry, in spinel-type anode materials, Co, Mn, and Ni work on the basis of reversible conversion reaction[12,22]. Sn and Zn can undergo pure alloying with Li, or operate using both mentioned Li-storage mechanisms reversibly[4,5,34]. Metallic Mg also can be reversibly lithiated (forming Li-Mg intermetallics)[35], however, in a form of oxide it is typically electrochemically inactive[13,36]. Interestingly, it has been recently reported that in $(Co_{0.2}Cu_{0.2}Mg_{0.2}Ni_{0.2}Zn_{0.2})O$ HEO the lithiation kinetics of Li-Mg reaction can be catalyzed, making such mechanism possible[17]. Consequently, the initially proposed $Sn(Co_{0.2}Mg_{0.2}Mn_{0.2}Ni_{0.2}Zn_{0.2})_2O_4$ composition combines an advantageously high amount of alloying-based elements (i.a. in terms of high capacity and low operating voltage), together with the elements reacting with Li by means of conversion reaction (thus ensuring buffering Li_2O matrix and conductive network of metallic particles). At the same time, multiple components in the equimolar ratio shall result in enhanced cycling performance, typical for HEO-based electrodes.

Supplementary Note 4: Phase analysis of Sn1-ME5 and Sn0.8-ME5

Regarding the Sn1-ME5 sample, there is ca. 93 wt% of the spinel phase and 7 wt% of the SnO_2 secondary phase, according to the Rietveld analysis (not shown here, assuming that the impurity is tetragonal SnO_2). For the conventional solid-state synthesized tin spinels, in some cases, the additional step of synthesis including milling and further sintering stages, improves the phase purity[33,37]. However, as presented in Figure S1a, such a procedure did not significantly change the content of SnO_2 in the sample. Based on XPS measured for Sn0.8-ME5 (see the description in Supplementary Note 5 below) it is documented that Mn tends to exhibit an oxidation state higher than +2 (while Sn is at +4 state, based on XPS and Mössbauer spectroscopy, Supplementary Note

5). As a consequence, it leads to the precipitation of SnO_2, making synthesis of the tin-stoichiometric HEO impossible.

On the other hand, the content of rock salt-structured $A^{2+}O$ secondary phase detected through XRD for Sn0.8-ME5 material (Figure S1b) is significantly lower compared with SnO_2 amount in Sn1-ME5. Moreover, after 2nd sintering the impurity peak intensity is decreased, suggesting that it might originate from the residues of precursor oxides used for the solid-state route, and can be further incorporated into the spinel structure after additional heat treatment.

Importantly, SnO_2 impurity in Sn1-ME5 leads to substantially deteriorated electrochemical performance (Table S1), most likely due to the formation of aggregated metallic Sn grains and significant volume changes during work in a cell[38–40]. Considering the minor amount of rock salt-type secondary phase in Sn0.8-ME5 sample, it can undergo conversion reaction upon lithiation forming nanoparticles, as documented by the disappearance of peaks from this phase during the 1st cycle in a half-cell visible through operando XRD measurements (see Figure 3a,b). To conclude, rock salt-type secondary phase does not have considerable influence on the electrochemical parameters. Consequently, for all further studies, we selected the sample after single-step preparation process, in order to keep the synthesis method as simple as possible.

Table S1. Electrochemical parameters of the electrode with Sn1-ME5 material cycled in half-cell in the voltage range of 0.01-2.5 V.

Specific current [mA g^{-1}]	Discharge specific capacity after 2nd cycle [mAh g^{-1}]	Discharge specific capacity after 50th cycle [mAh g^{-1}]	Capacity retention after 50 cycles (compared with 2nd cycle) [%]
200	562	230	41

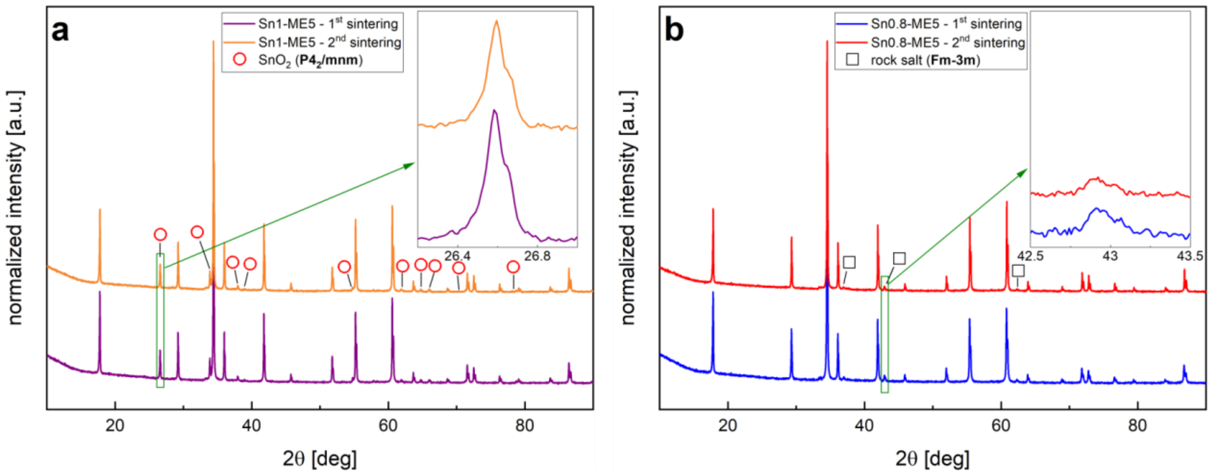

Figure S1. XRD data for Sn0.8-ME5 and Sn1-ME5 after 1st and 2nd sintering: a) Patterns for Sn1-ME5 with zoomed most intense reflection for SnO_2 secondary phase (inset); red circle symbol corresponds to the cassiterite phase ($P4_2$/mnm). The normalized intensity scale on the insets is the same. b) Patterns for Sn0.8-ME5 with zoomed most intense reflection for rock salt-structured secondary phase (inset); black square symbol corresponds to the rock salt phase (Fm-3m).

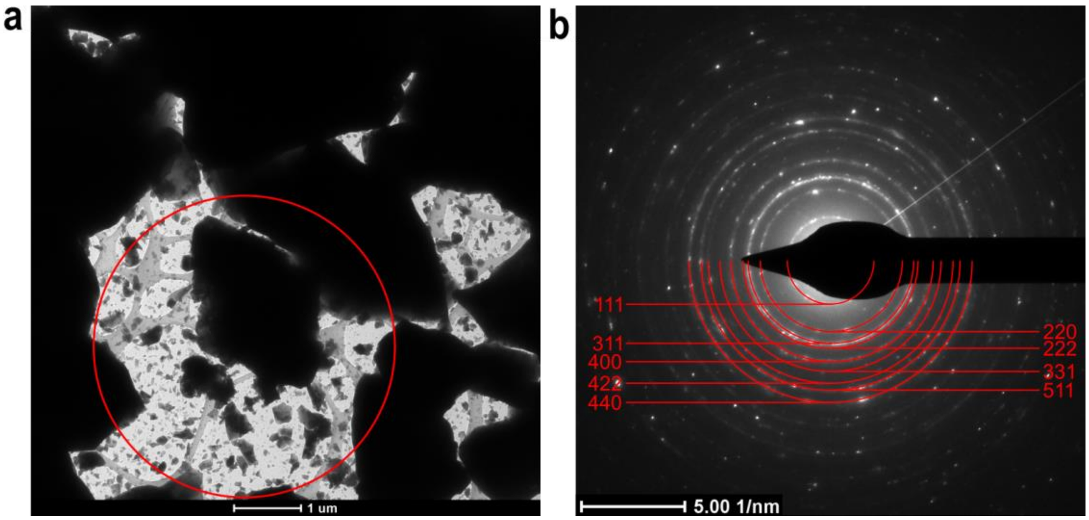

Figure S2. Bright-field TEM image for Sn0.8-ME5 powder with corresponding SAED pattern: a) TEM image, showing large grains of the manually ground pellet. b) Selected area electron diffraction (SAED) pattern from the region marked in (a) with rings assigned to the spinel structure (Fd-3m).

Supplementary Note 5: Structural properties of Sn0.8-ME5 as derived from Raman, Mössbauer and X-ray photoelectron spectroscopies

Raman spectroscopy

The measured spectrum for the $SnZn_2O_4$ reference material (red line in Figure 1b) is in good agreement with previous literature reports[41,42]. Based on that, the bands can be assigned to five Raman-active vibrational modes: $3F_{2g}$, E_g, and A_{1g}, as predicted by the group theory for the Fd-3m space group[43,44]. Regarding the spectra collected for Sn0.8-ME5 HEO (black line in Figure 1b), we have found minor differences depending on the location on the sample, likely originating from slightly different local occupancy of cations in the spinel lattice[44], even though the extent of this effect was rather limited. Nevertheless, for this reason, the presented Raman spectrum is an average from 10 measurements in different positions on the sample's surface. As can be seen in Figure 1b, the positions of the respective peaks for the HEO composition are shifted toward the smaller values of Raman shift, which can be associated with the change in interatomic force constants, likely resulting from the change of ionicity of the bonds. Still, probably the most profound difference between spectra measured for the HEO and $SnZn_2O_4$ are much broader peaks for the former composition, which originates from the emergence of new, unpredicted Raman-active modes and their subsequent overlapping[43,45,46]. It should be underlined that due to this overlapping and significant broadening, it is not possible to precisely determine the number and position of all of the bands in a process of deconvolution. However, by comparison with the literature data for both HEO[44,47] and conventional spinels[43,45], as well as measured spectrum for the $SnZn_2O_4$, selected peaks can still be assigned to the five Raman modes expected to occur for the spinel phase (Figure 1b), proving that the HEO exhibits the inverse spinel structure. In general, the emergence of new bands for conventional spinel oxides is commonly known and widely discussed in the literature[43,45,46,48]. Nevertheless, for the studied HEO, this phenomenon, appearing on the spectrum as asymmetric peaks characterized by large widths, is much more pronounced than for conventional materials, which can be explained by means of three different effects. First of all, it can be associated with high cation disorder in the high entropy spinel lattice, due to multiple possible combinations of the elements within the 2nd coordination sphere, similarly as in the two-component spinels (e.g. $NiAl_2O_4$)[43,45,46], but to a larger extent. This is especially visible by formation of the sub-bands near the A_{1g} symmetry band at Raman shifts around 600 cm^{-1} [43–45]. Since the A_{1g} mode can be related to the elemental distribution within the octahedral sites[45], the observed effect can be correlated with the existence of distinct octahedral units in the lattice, occupied by multiple cations introduced by the high entropy approach[44,47]. As a consequence, each configuration might exhibit slightly different energy for this particular vibrational mode, creating additional bands[49], which would be absent in the simpler compositions, such as the reference $SnZn_2O_4$. Another phenomenon leading to the emergence of new Raman bands in spinels is the so-called Jahn-Teller effect, typical e.g. for spinel oxides containing Mn^{3+} or Ni^{2+} [45,49], and previously observed in other HEOs[50,51]. Both Mn^{3+} and Ni^{2+} are present in the Sn0.8-ME5 material, as indicated by the XPS studies (Figure S3c and the description below). The possible Jahn-Teller distortions, leading toward lower

tetragonal symmetry of the lattice, can relax the Raman spectroscopy selection rules and cause activation of new bands[52,53]. It should be stressed that this is likely a local, limited effect, as the lower symmetry was not detected with XRD measurements. Finally, the last phenomenon resulting in the formation of new bands, somewhat related to the previous one, is a loss of degeneration of some of the vibrational modes[49,53]. E_g and F_{2g} modes are doubly and triply degenerated, respectively. Local breaking of symmetry (i.a. due to the Jahn-Teller effect described above) can result in a loss of degeneration and the presence of new bands near the original positions of the initially degenerated modes[49,53]. This is prominent for the F_{2g} bands in the spectrum for HEO. It is not clearly seen for the E_g mode due to the low intensity and high width of this band. In the end, it should be mentioned that there are some discrepancies in the literature regarding the origins of the A_{1g} band in spinels (whether it is related more to octahedral or tetrahedral sites[43,49]). Assuming that this band is related to tetrahedral units, the appearance of A_{1g} subbands could be related to the transfer of some amount of Sn to tetrahedral sites[33], going toward a random spinel, similarly as suggested by the Rietveld refinement of the measured XRD pattern. Overall, the Raman spectroscopy studies indicate that the studied HEO exhibits severely distorted complex inverse spinel structure with a high level of cation mixing and disorder within the crystal lattice.

Mössbauer spectroscopy

Mössbauer spectra of Sn0.8-ME5 and $SnZn_2O_4$ samples are shown in Figure 1e and S3a, respectively. Even though they appear to be wide, single lines, the analysis indicates that they are doublets, with a bilinear structure not clearly visible due to the broad natural linewidth of ^{119}Sn. Analysis of such spectra must take into account their potential deviation from symmetry, which may be due to the multiphase nature of the sample or the presence of tin at different crystal positions. Therefore, the spectra were analyzed in terms of the quadrupole splitting distributions (QSD) with a possible QS(IS) relationship. However, such relationship was extremely weak and the symmetry of the spectra is very high. The QSD curves for both Sn0.8-ME5 and $SnZn_2O_4$ samples are single peak with a slight tail toward the higher QS values. Importantly, they are qualitatively very similar, proving that the interactions responsible for their formation are also similar. Since the same fit quality can be achieved more simply by using one quadrupole doublet instead of the QSD, only the former approach has been used. The good fit quality is confirmed by the difference spectra, which can be considered purely a statistical noise. The extracted isomer shifts (IS), quadrupole splitting (QS) an linewidths (G) are collected in Table S2. For the reference sample, the spectrum is in good agreement with the literature data for solid-state synthesized $SnZn_2O_4$ [33]. Both IS and QS values are slightly higher than previously reported[33],which likely originates from differences in the synthesis method. Both IS and QS describing the HEO spectrum are distributed due to significant lattice (polyhedra) distortion caused by a random distribution of cations in the second coordination sphere[35,40,41], which is in line with the findings from Raman spectroscopy. When comparing the spectra for Sn0.8-ME5 and $SnZn_2O_4$, it should be remembered that the changes of Sn neighbors may lead to either increase or decrease of QS, which result in more impact on the QS distribution (Figure S3a), and less influence on the average QS value given in Table S2. Also, the observed relatively small difference in QS values between samples is related

to the fact that cations in the considered oxides are far from each other, additionally separated by oxygen ions. It is worth mentioning that the observed severe lattice distortion can be assigned into one of the so-called high entropy effects[54], also documented for other HEOs using Mössbauer spectroscopy[55,56]. Unfortunately, due to the poor structure of the spectrum, the effects of these distributions are practically indistinguishable and are manifested primarily by an increase of QS and G. The average IS value for Sn0.8-ME5 is also noticeably higher than for $SnZn_2O_4$, which most likely originates from a different type of second neighbors introduced in the high entropy lattice (distinct chemical environment)[57–59]. The IS and QS values for HEO indicate that Sn is exclusively at +4 oxidation state, similarly to the reference sample[33,60,61]. Some literature reports also suggest that such an increased average QS could be related to the presence of several overlapping doublets, again emerging from lattice distortions, but also possibly from the location of some part of Sn^{4+} in tetrahedral positions in the spinel structure[33,59,62]. However, because of the very high symmetry of the spectra the first option (distortion) seems to be more probable.

X-ray photoelectron spectroscopy

We utilized the XPS technique to establish the oxidation states of the components in the spinel-type Sn0.8-ME5 HEO. The results are presented in Figure S3b, showing the high-resolution spectra for Sn, O, Mn, Co, Ni and Zn. There is also evidence on the survey scan (not shown here) of Mg presence, but due to the overlap of both Mg 2p and Mg 2s lines with other intense peaks from Mn (3s, 3p) and Zn (3p), the quantitative and qualitative analysis is ambiguous and burdened with a large error. However, as expected (and further proven by the performed XAS measurements, see Figure 1f), Mg exhibits +2 oxidation state. The Sn $3d_{5/2}$ spectrum was fitted with a single line centered at 486.4 eV. It can be assigned to the existence of either Sn^{2+} or Sn^{4+} oxidation state[63–65]. However, combined with the Mössbauer spectroscopy results (Figure 1e, S3a), it can be stated that there is only Sn^{4+} in the sample. The O 1s spectrum was fitted with three components: the first main line centered at 529.9 eV, which points out the existence of lattice oxygen; the second line (minor) centered at 531.5 indicates the presence of either defective oxygen in metal oxides or organic species (O=C) from contamination; the third line (minor) found at 532.9 eV originates either from -OH and/or C-O type bonds from organic contamination[64,66]. Spectrum collected at Mn $2p_{3/2}$ region was fitted with five components with the first line centered at 640.7 eV, indicating the existence of Mn^{3+} oxidation state like in the Mn_2O_3 [67]. The four lines within the energy range of 642-647 eV are due to the multiplet splitting phenomena. The Co $2p_{3/2}$ spectrum was fitted with four components with the first line centered at 780.4 eV, proving the existence of Co^{2+} as in CoO or $Co(OH)_2$ [68,69]. Three lines within the energy range of 783-789 eV are due to the multiplet splitting phenomena. Spectrum collected at Ni $2p_{3/2}$ region was fitted with four components with the first line centered at 855.1 eV related to the existence of Ni^{2+} like in $Ni(OH)_2$ [67,70]. Three lines within energy the range of 858-864 eV are due to the multiplet splitting phenomena. At the Zn $2p_{3/2}$ region, the spectrum was fitted with the single line centered at 1021.2 eV, which indicates the existence of Zn^{2+}, similarly to the ZnO [66,68].

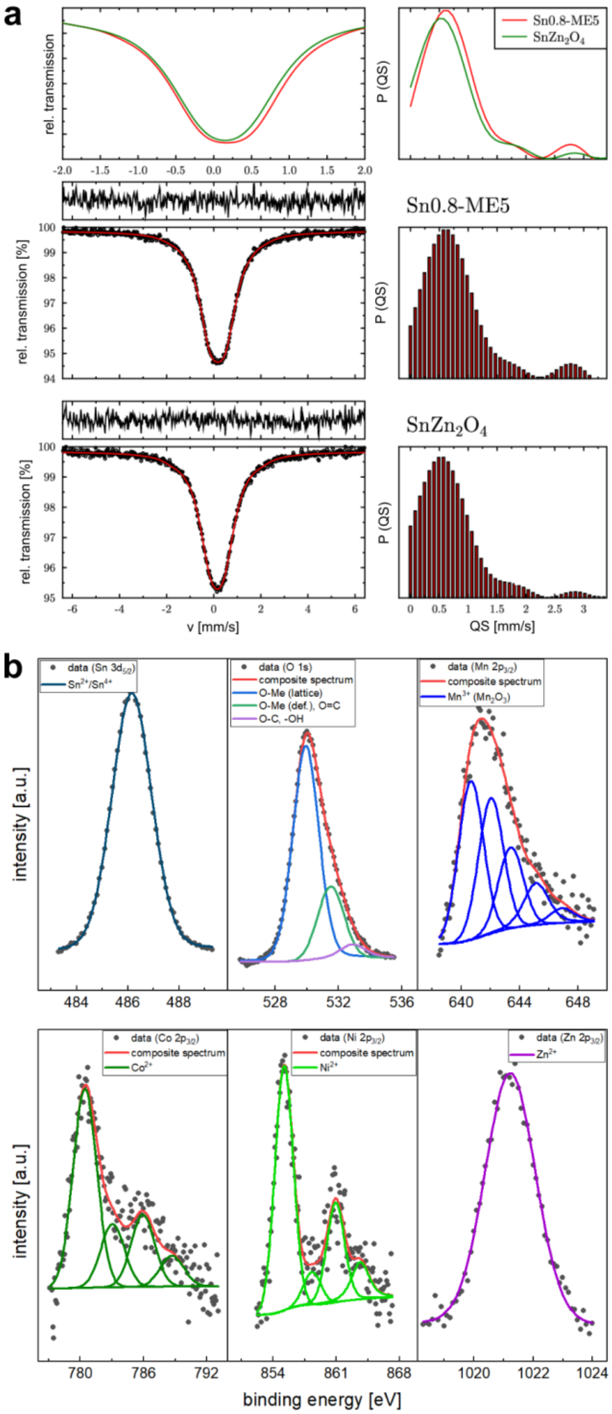

Figure S3. Mössbauer and X-ray photoelectron spectroscopy results: a) Room temperature ^{119}Sn Mössbauer spectrum for $SnZn_2O_4$ reference material (at the bottom) fitted assuming one quadrupole doublet with the calculated parameters presented in Tab S2 and difference spectrum, together with quadrupole splitting distribution (QSD) analysis. Above, for a better comparison, there is the spectrum for the Sn0.8-ME5 sample, and both spectra presented collectively on the same graphs. b) Fitted high-resolution XPS spectra for Sn0.8-ME5 sample; references used for fitting are described in the text.

Table S2. Isomer shift (IS) and quadrupole splitting (QS) values from fitted Mössbauer spectra.

Sample	Isomer shift [mm/s]	Quadrupole splitting [mm/s]	Line width [mm/s]	Area [%]
Sn0.8-ME5	0.174(6)	0.628(8)	1.17(2)	100
$SnZn_2O_4$	0.148(6)	0.559(9)	1.16(2)	100

Supplementary Note 6: Morphology and chemical composition analysis of Sn0.8-ME5

The SEM micrograph of the manually ground pellet of Sn0.8-ME5 material (Figure S4a) shows irregular grains with different sizes, varying from below 1 μm up to even 10 μm. Additionally, the particle size distribution measured using the dynamic light scattering method (DLS) is presented in Figure S4c, showing a generally bimodal distribution, with two maxima at ca. 2 and 10-30 μm. The fraction of particles above 10 μm observable in DLS studies likely originates from the presence of agglomerates. Average chemical composition analysis performed for the pellet's cross-section (Table S3) show that the composition is close to the nominal one, within the accuracy of the semi-quantitative EDS method (the targeted composition is presented also in Table S3). The content of Mg is somewhat lower than expected, however, this effect is likely exaggerated due to the fact that it is a light element, especially compared to the rest of the cations. Likewise, the content of the heaviest element, Sn, is probably overestimated. Results of point analysis in both SEM and STEM modes (selected points are marked in Figures S4a,b) are presented in Table S3. The established composition for the grains of the spinel phase is again close to the targeted one, especially considering STEM/EDS technique characterized by higher spatial resolution. The exemplary measurement for the minor rock salt-structured impurity grain, detected via XRD method (Figure S1b), indicates that this phase is composed mostly of Ni, Mg, and Co (single oxides of these elements crystallize in the rock salt structure), while depleted with Sn, Mn and Zn.

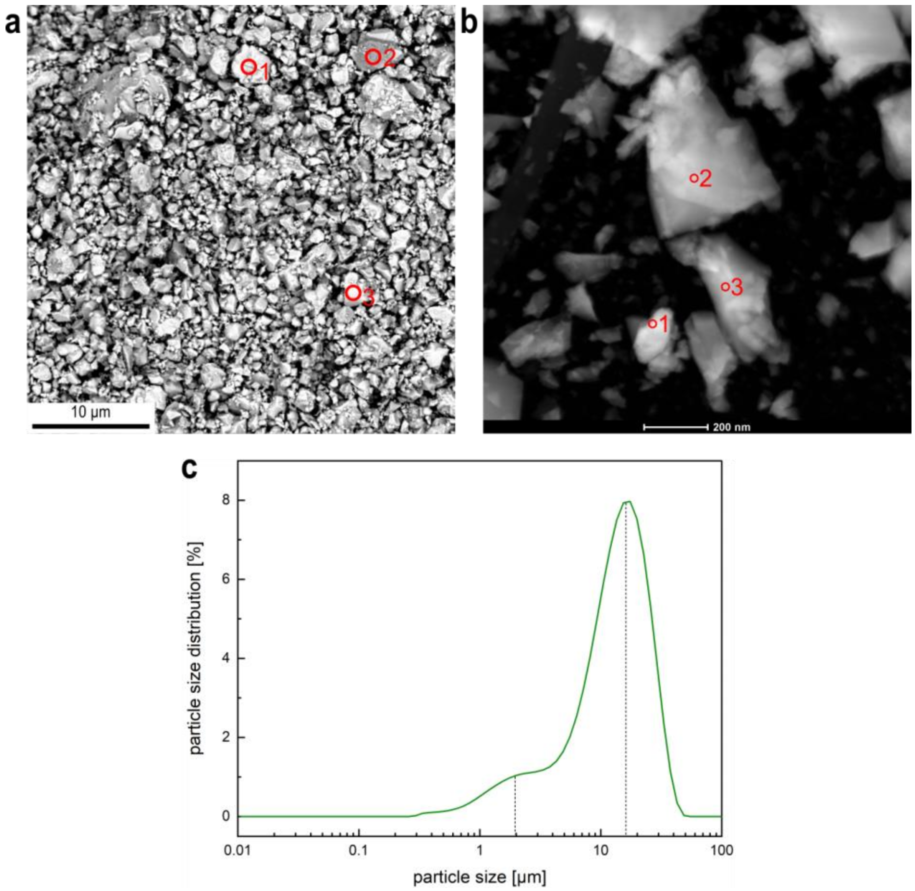

Figure S4. Structure and chemical composition of Sn0.8-ME5 powder: a) SEM image with marked points of EDS analysis (results are presented in Table S3). b) STEM image with marked points of EDS analysis (results are presented in Table S3). c) Particle/agglomerate size distribution measured through DLS method.

Table S3. Chemical EDS analysis. Average chemical compositions measured through EDS area analysis (mean) and EDS point analysis in microscale (using SEM, corresponding points in Figure S4a) and nanoscale (using STEM, corresponding points in Figure S4b) for the considered HEO, together with targeted cations content according to the initially assumed composition.

**SEM/EDS analysis**	**Cations content [at. ratio]**					
	**Sn**	**Co**	**Mg**	**Mn**	**Ni**	**Zn**
Sn0.8-ME5, mean from map	0.319	0.144	0.109	0.154	0.124	0.150
Sn0.8-ME5, 1st sintering, point 1	0.316	0.123	0.146	0.145	0.131	0.140
Sn0.8-ME5, 1st sintering, point 2	0.007	0.241	0.271	0.011	0.414	0.06
Sn0.8-ME5, 1st sintering, point 3	0.326	0.150	0.145	0.145	0.101	0.132
**STEM/EDS analysis**	**Cations content [at. ratio]**					
	**Sn**	**Co**	**Mg**	**Mn**	**Ni**	**Zn**
Sn0.8-ME5, 1st sintering, point 1	0.244	0.161	0.162	0.148	0.118	0.168

Sn0.8-ME5, 1st sintering, point 2	0.262	0.152	0.122	0.149	0.141	0.173
Sn0.8-ME5, 1st sintering, point 3	0.253	0.166	0.149	0.147	0.121	0.163
	**Target cations content [at. ratio]**					
	**Sn**	**Co**	**Mg**	**Mn**	**Ni**	**Zn**
Sn0.8-ME5	0.267	0.147	0.147	0.147	0.147	0.147

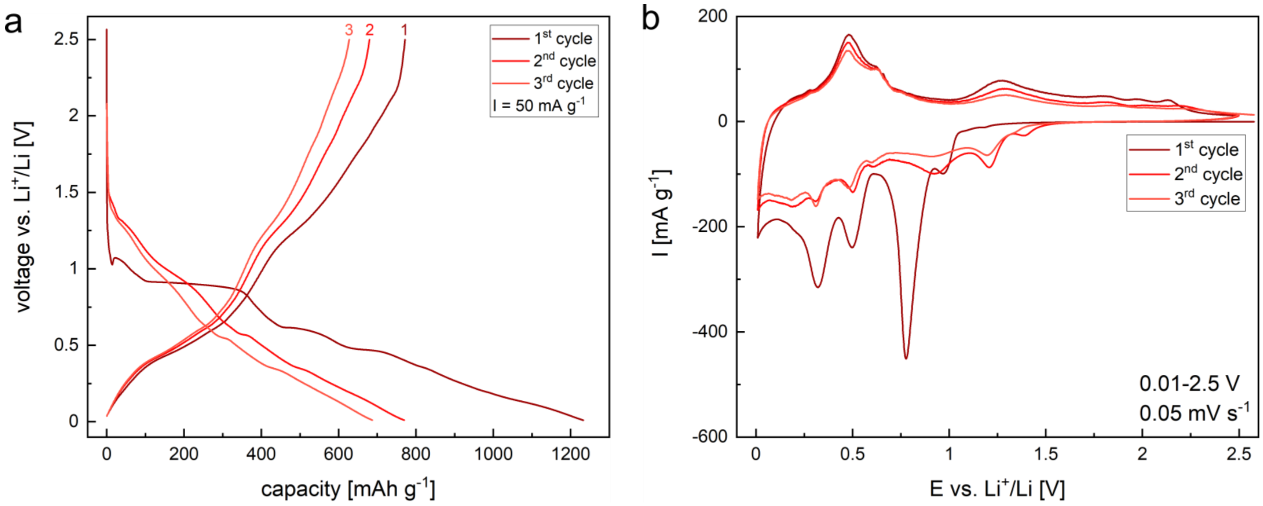

Figure S5. Electrochemical properties of the precursor for HEO synthesis (ball-milled mixture of SnO_2, Co_3O_4, MgO, MnO, NiO, and ZnO oxides): a) GDC curves for the electrode with PVDF binder in the voltage range of 0.01-2.5 V under the specific current of 50 mA g^{-1} for three initial cycles; Numbers on top indicate cycle number; There is a small, unfavorable lag phenomenon visible at ca. 1.0 V. b) Cyclic voltammetry curves for three initial cycles in the voltage range on 0.01-2.5 V with the scan rate of 0.05 mV s^{-1}. The behavior down to ca. 0.8 V can be interpreted as related to the formation of the SEI, as it does not reappear on the subsequent cycles.

Supplementary Note 7: Reasons for residues of the spinel phase in the operando XRD measurements

The low-intensity peaks from the Fd-3m spinel phase remaining after the decomposition (constant intensity with the time, Figure 3a) are due to limitations of the used operando cell. The comparison of XRD patterns for the fully lithiated electrode in the operando cell and a coin cell (ex-situ measurement) is presented in Figure S6. As visible, there are no peaks from the spinel phase observed for the electrode discharged in the coin cell. Therefore, it can be reasonably stated that the residue peaks in Figure 3a come from unreacted pristine Sn0.8-ME5, as not the entire volume of the electrode is active in the operando cell.

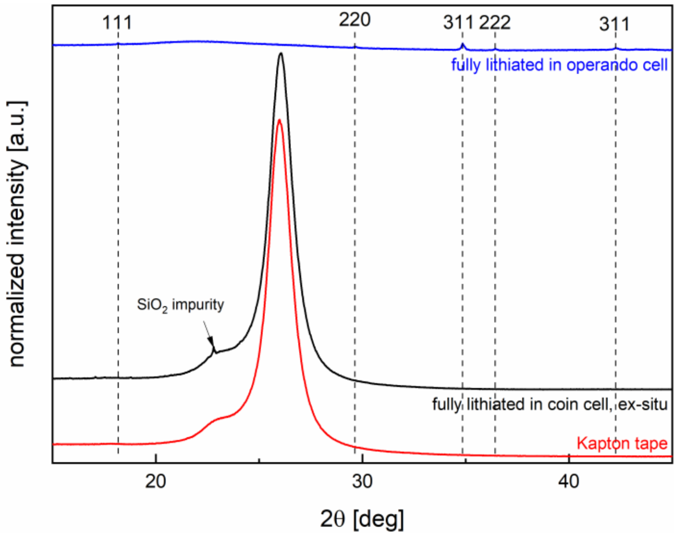

Figure S6. Comparison of operando and ex-situ XRD patterns of fully lithiated Sn0.8-ME5. Blue pattern corresponds to fully lithiated material from operando cell; Black pattern is for fully lithiated Sn0.8-ME5 electrode measured ex-situ, sealed in a glovebox using Kapton tape (additional peak likely originates from SiO_2 impurity from XRD holder); Red XRD pattern corresponds to reference measurement of Kapton tape. Miller indices correspond to the Fd-3m spinel phase.

Supplementary Note 8: Operando EIS and DRT analysis

All the measured EIS spectra are presented in Figure S7a (lithiation) and Figure S7b (delithiation). Additionally, for the exemplary (de-)lithiation states of the half-cell (fresh and fully discharged) the spectra are presented with marked characteristic frequency points on the Nyquist plots (Figure S7c,d). Since DRT methodology can be applied only for the part of the spectrum converged toward the real axis[71,72], results were analyzed in 1-10^6 Hz frequency range, which does not involve Warburg diffusion part of spectra (see the frequency values marked on the exemplary spectra in Figure S7c). To perform reliable DRT, the EIS data must be compatible in terms of Kramers-Krönig (KK) relations[72,73]. For this reason, all the spectra were tested using Lin-KK Tool[74], giving low residual values for both real and imaginary parts at least down to the frequency of 1 Hz. Consequently, the DRT methodology could be applied in the studied frequency range (Figure 3c). To help the assignment of the respective peaks, we measured EIS for a symmetrical Li-Li coin cell (Figure S7e), with the DRT shown in Figure S7d. Starting with the highest frequencies for Sn0.8-ME5 half-cell in Figure 3c, at ca. 10^5 Hz a minor, practically constant peak is visible, which can be assigned to the contact impedance[71,72]. In general, electrochemical reactions do not occur at such high frequencies. There are two another peaks at frequencies ca. 10^4 Hz and at around 10^2-10^3 Hz for each (de-)lithiation state, with the latter one being the most intense peak overall. These peaks are also observed in the DRT analysis result for the symmetrical Li-Li cell (Figure S7f), which is in good agreement with the literature[71,72]. Therefore, they can be correlated with polarization effects from SEI and charge transfer connected with the Li counter electrode, as well as SEI of the

Sn0.8-ME5-based electrode[71,72]. Each of these phenomena influences the recorded signal, however, they create overlapped peaks with two maxima present, but which cannot be further separated even with the DRT analysis. It is worth noting that P_{SEI} are constantly decreasing with time during both discharge and charge (lower resistance), as well as are shifted toward higher frequencies (better kinetics). Hence, the unfavorable effect of the unstable SEI, observed e.g. for Si and SiO[72], is not detected for the Sn0.8-ME5 active material. The most prominent changes with (de-)lithiation stage can be observed for the family of P_{CT} peaks, which show even up to 3 maxima. Based on the literature[71,72], as well as the significant dependence on the state of charge, P_{CT} are mainly related to the charge transfer processes in the Sn0.8-ME5 electrode.

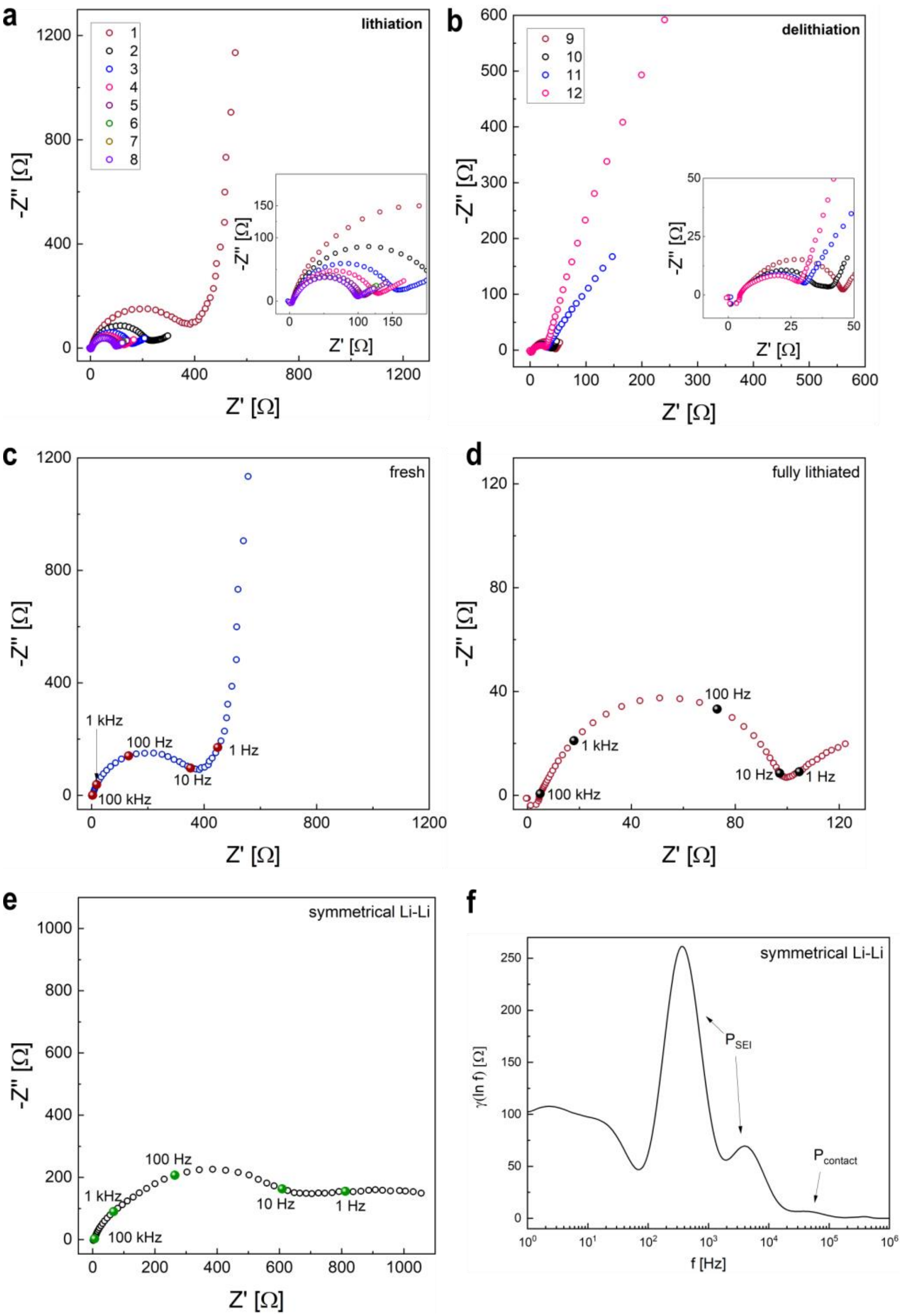

Figure S7. EIS and DRT analysis: a,b) Raw EIS data for the half-cell with Sn0.8-ME5 active material measured at different (de-)lithiation states marked with numbers corresponding to the GDC curves presented in Figure 3c during lithiation (a) and delithiation (b). c,d) Selected EIS spectra with marked frequency points measured at open circuit voltage (fresh cell) (c) and fully lithiated cell (d). e,f) EIS spectrum for symmetrical Li-Li cell (e) with performed DRT analysis (f) with assignment of peaks based on [71,72].

Supplementary Note 9: Analysis of Mössbauer and XAS spectra measured at different stages of (de-)lithiation

Ex-situ Mössbauer spectroscopy

The spectra for the lithiated and delithiated Sn0.8-ME5 (Figure 4b) clearly shown two separate components. Unfortunately, a full quantitative analysis of the data could be performed only in a limited range, due to the relatively weak signal. The spectra were analyzed assuming the existence of two quadrupole doublets, and the obtained IS and QS parameters (Table S4) clearly indicate the presence of Sn^0 and Sn^{4+} [60,75]. Interestingly, high value of IS for the Sn^0 in the lithiated state hints low level of lithiation, as highly lithiated Li-Sn intermetallics (e.g. $Li_{22}Sn_5$) generally yield smaller isomer shifts[60,61,75,76]. Within the experimental error, the results obtained for the spectra of both samples are similar. However, it can be noticed that the spectrum of the fully lithiated sample is characterized by slightly higher IS and QS values, which can be qualitatively explained by the greater influence of non-Sn nearest neighbors on hyperfine parameters in the structure of this sample.

Table S4. Isomer shift (IS) and quadrupole splitting (QS) values from fitted Mössbauer spectra for fully lithiated and delithiated electrodes.

Sample	Isomer shift [mm/s]	Quadrupole splitting [mm/s]	Line width [mm/s]	Area [%]	Assignment
Sn0.8-ME5 lithiated	0.56(2)	0.50(12)	1.44(16)	71(6)	Sn^{4+}
	2.45(3)	0.50(06)	0.81(13)	29(6)	Sn^0
Sn0.8-ME5 delithiated	0.48(2)	0.42(13)	1.32(15)	66(6)	Sn^{4+}
	2.35(3)	0.34(12)	1.12(25)	34(6)	Sn^0

Ex-situ X-ray absorption spectroscopy

To study composition, local geometry and chemical states variations of Mn, Co, Ni, Zn, Mg in Sn0.8-ME5 HEO, XAS measurements were performed at different states of charge in PFY mode. XANES spectra of the Mn, Co, Ni, Zn L_3- and L_2-edges and Mg K-edge are presented in Figure 4a and Figure S8.

During the lithiation, L-edges of Mn are slightly moved toward lower energy compared to the pristine sample, and the intensity of peaks at energy 642.1 eV and 643.6 eV significantly decreases, which is related to the manganese reduction process. At the end of lithiation process, L-edges position corresponds to Mn^0, according to the references[77], but the broad and asymmetrical shoulder proves the presence of residual of Mn^{2+} [78]. After full delithiation, the L-edges shift toward

higher energy, indicating oxidation of Mn^0. Since the intensity of peaks at 642.1 eV and 643.6 eV for delithiated sample is significantly lower than for the pristine one, it seems that the valency of manganese returns to +2 after the 1st cycle. After 20 cycles only Mn^0 is observed for the fully lithiated sample. Thus, besides the initial cycle, manganese changes the valency from +2 to 0 and back for delithiation and lithiation processes, respectively.

Regarding spectra for Co L_3- and L_2-edges, for the fully lithiated sample the main peak is slightly shifted to 778.6 eV and the extreme at 779.9 eV disappears. The measured spectrum is in good agreement with the reference Co^0 one. However, an asymmetrical tail could indicate some residual $Co^{2+/3+}$. After the 1st cycle cobalt is present in the lithiated and delithiated samples mostly in the 0 oxidation state, and it remains practically electrochemically inactive.

For the Ni XANES spectra, the right peaks of both L-edges decrease as lithiation is proceeding and the spectrum corresponds to Ni^0 reference after full lithiation[79]. Then, after delithiation, the right extreme of L_3-edge slightly increases, indicating that a small amount of Ni^0 is oxidized to Ni^{2+}. Only a small amount of Ni ions is involved in the oxidation and reduction processes during the subsequent cycles, but Ni remains mostly electrochemically inactive.

For the measured L_3 edge of Zn, after partial lithiation to the capacity of 340 mAh g^{-1} intensity of peaks decreases. From the sample lithiated to 630 mAh g^{-1} the L_3 edge is shifted toward lower energy due to the reduction to Zn^0 [80]. At the end of the 1st cycle (fully delithiated state) the L_3 edge moves back to higher energies and similar spectrum features as for pristine sample are observed. The higher intensity of pre-peak compared to the spectrum of the pristine sample indicates a different crystallographic environment of Zn^{2+}. Concluding, Zn is electrochemically active in Sn0.8-ME5 HEO by changing the valency from +2 after delithiation to 0 after lithiation.

In the case of spectrum for Mg K-edge, after partial lithiation the reduction of pre-peak intensity can be observed, which is related to the change in the crystallographic environment of Mg, while it still remains at +2 state. The K-edge is shifted towards lower energy after full lithiation, indicating a reduction of Mg^{2+} to Mg^0 [81]. However, the feature above 1323 eV is still observed, which corresponds to a residual Mg^{2+}. After full delithiation Mg K-edge moves back to higher energy similar to the pristine state, but still with a presence of Mg^0. It can be concluded that after 1st cycle the prevalent valence state of Mg is +2. The character of spectrum for the lithiated electrode after 20th cycle, while still suggests mixed valency of magnesium, indicates that there is even more Mg^{2+} than in the fully delithiated material after 1st cycle. Therefore, it seems that magnesium becomes partially disactivated on cycling. This may be related to the drop of the capacity during the initial cycles.

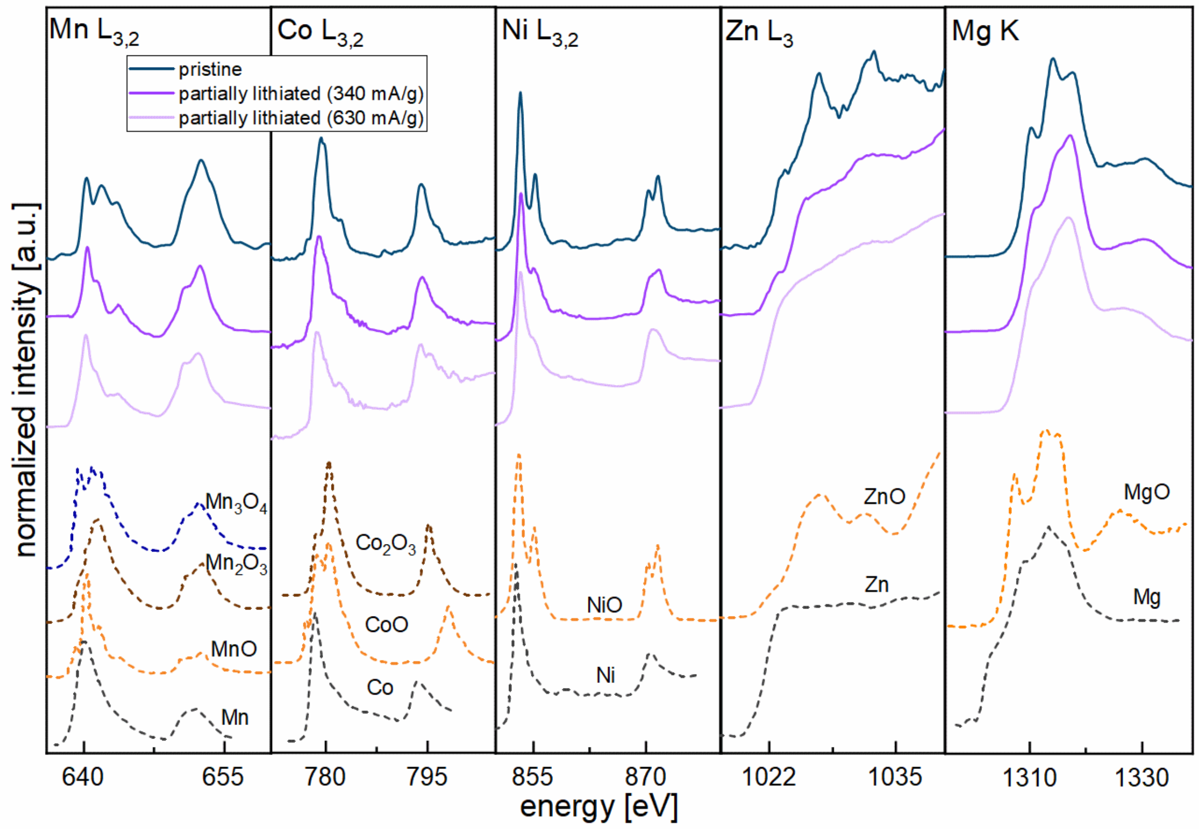

Figure S8. XAS of the partially lithiated Sn0.8-ME5. X-ray absorption near-edge structure spectra Mg K-edge and Mn, Co, Ni, Zn L_3- and L_2-edges measured in partial fluorescence mode (PFY) for pristine electrode, as well as for the electrodes lithiated to 340 mAh g^{-1} and 630 mAh g^{-1}, with the literature references[77–83] discussed in the text.

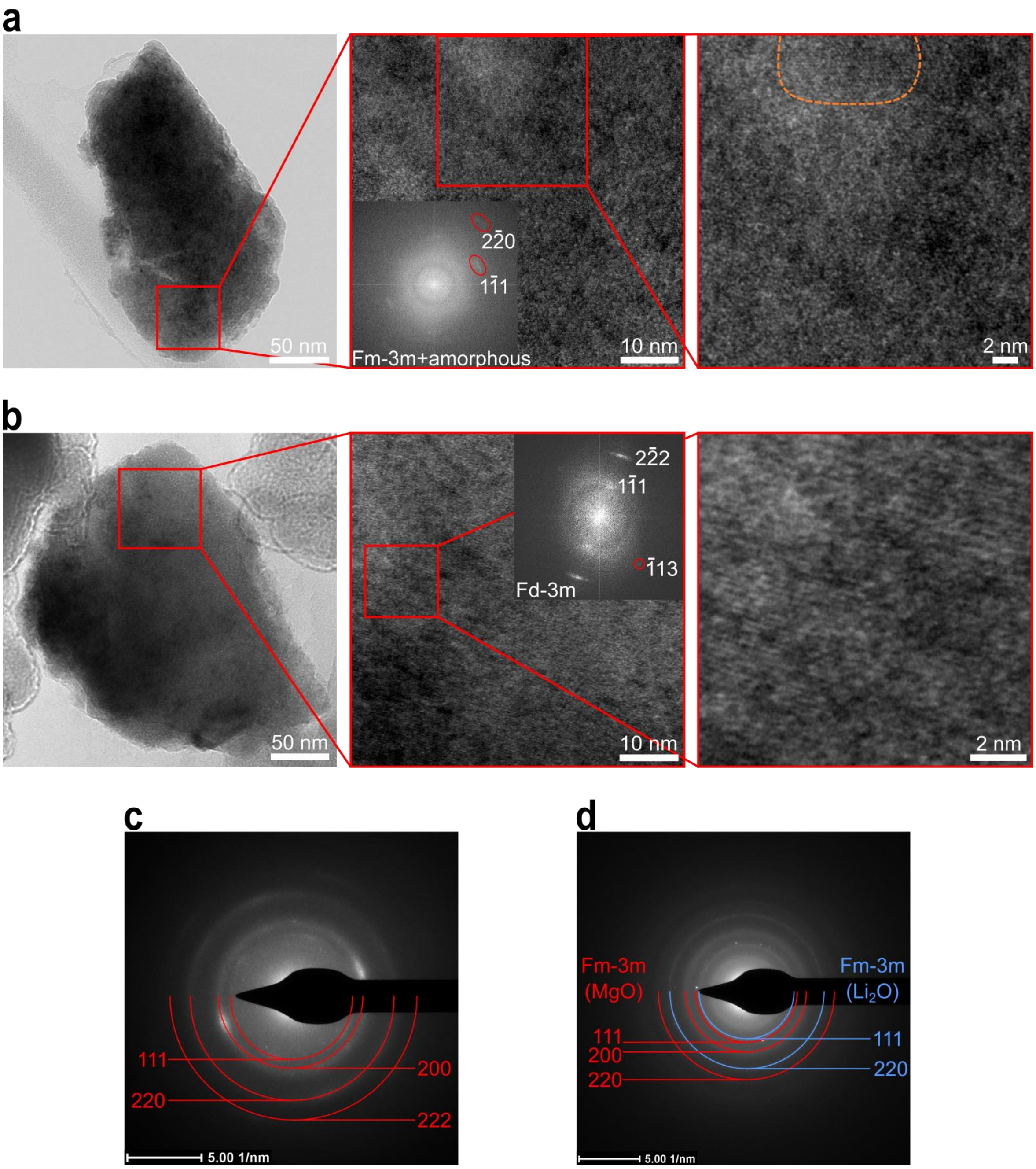

Figure S9. Ex-situ TEM studies of the fully lithiated Sn0.8-ME5: a) Bright-field TEM images with corresponding HR-TEM analysis and FTT pattern of the whole image, showing a mixture of rock salt (Fm-3m) and amorphous phases; In the zoomed HR-TEM image (right-hand side) orange dashed line distinguishes crystalline rock salt-type region from amorphous matrix. b) Bright-field TEM images with corresponding HR-TEM analysis and FTT pattern of the whole image, showing spinel-structured (Fd-3m) region. c) SAED pattern with marked rings and Miller indices assigned to rock salt-structured (Fm-3m) phase. d) SAED pattern with marked rings and Miller indices assigned to rock salt-structured phase (reference for MgO, red color) and Li_2O (blue color), which is typical product of conversion reaction.

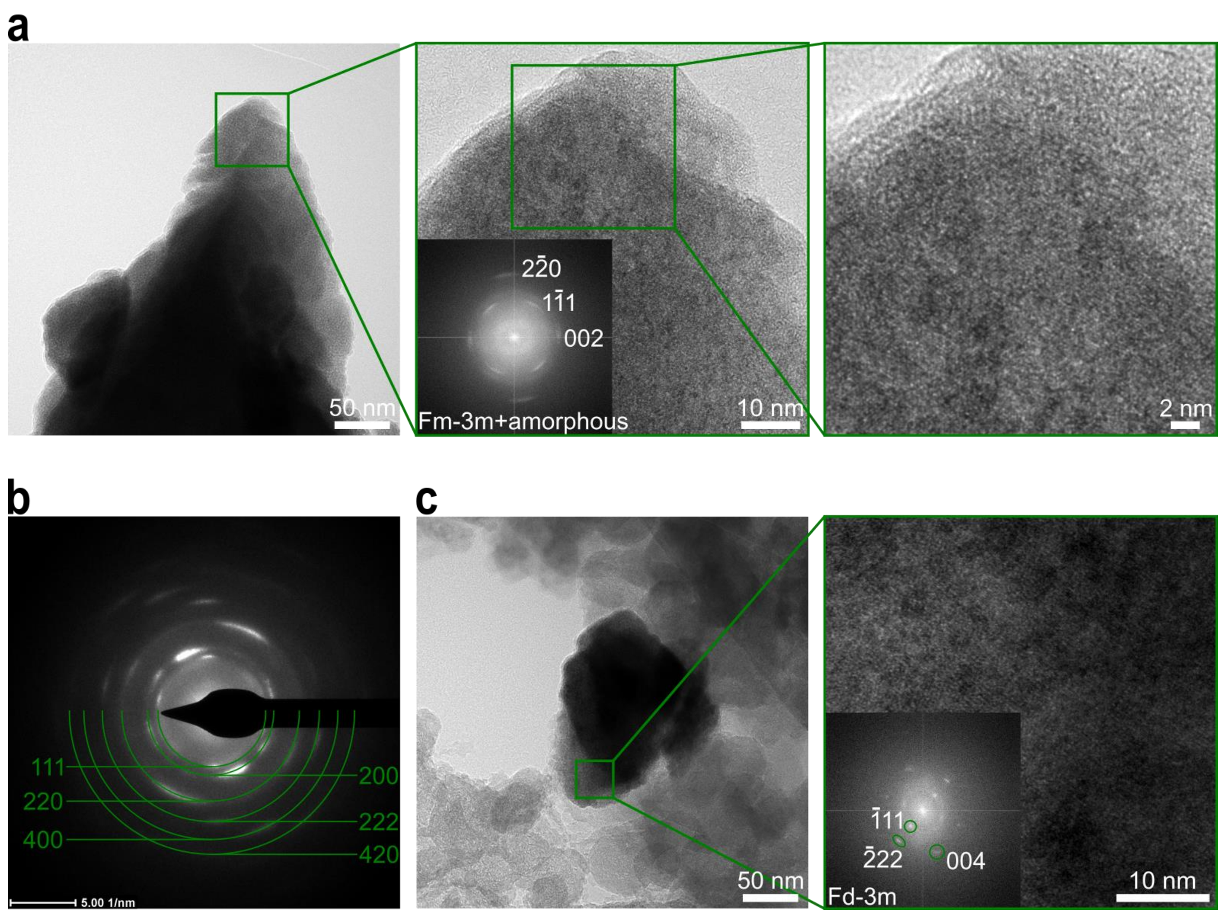

Figure S10. Ex-situ TEM studies of the fully delithiated Sn0.8-ME5: a) Bright-field TEM images with corresponding HR-TEM analysis and FTT pattern of the whole image, showing a mixture of rock salt (Fm-3m, higher level of crystallinity compared with the lithiated material) and amorphous phases. b) SAED pattern with marked rings and Miller indices assigned to the rock salt-structured (Fm-3m) phase. c) Bright-field TEM images with corresponding HR-TEM analysis and FTT pattern of the whole image, showing spinel-structured (Fd-3m) region.

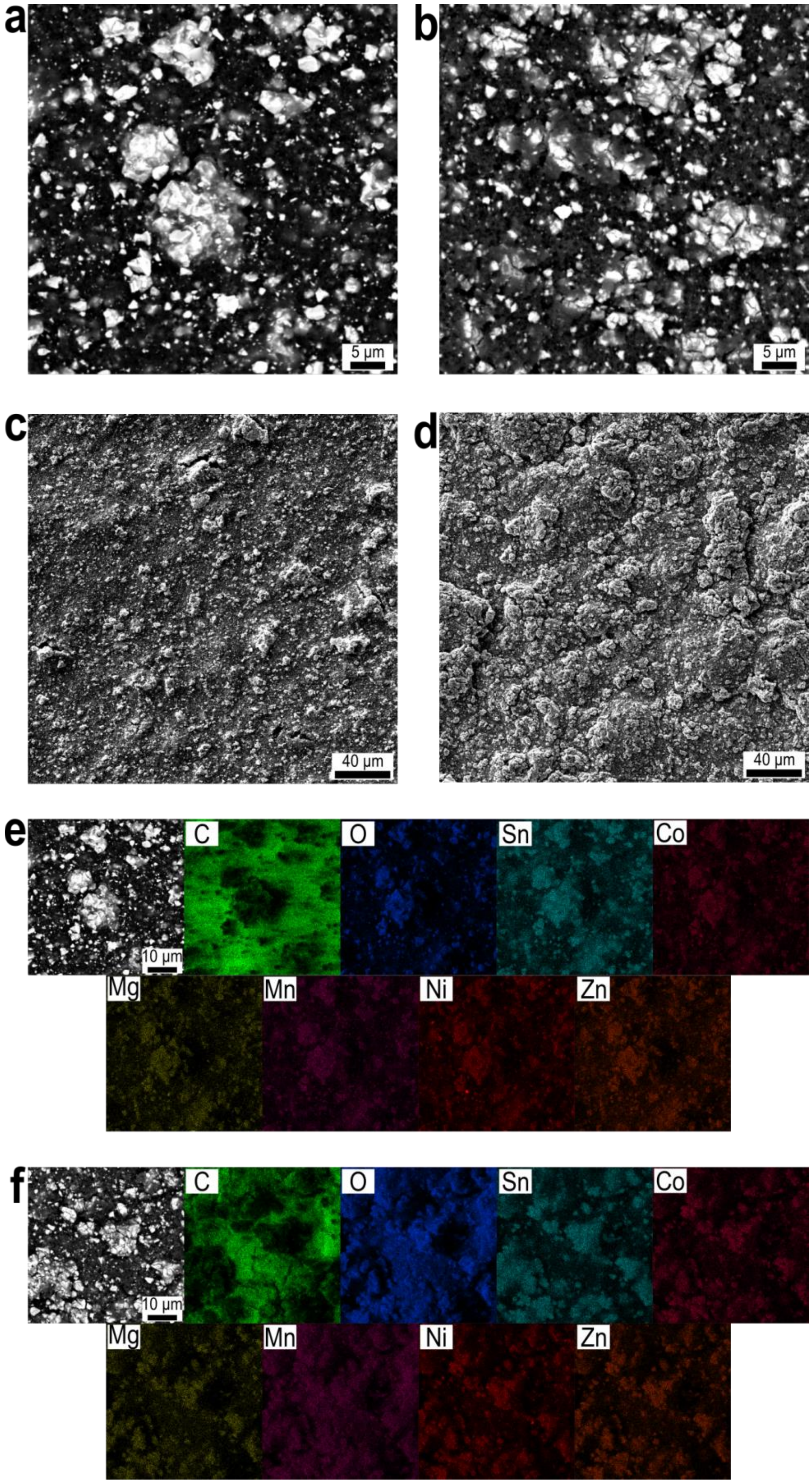
a
b
5 µm
5 µm
c
d
40 µm
40 µm
e
10 µm
C
O
Sn
Co
Mg
Mn
Ni
Zn
f
10 µm
C
O
Sn
Co
Mg
Mn
Ni
Zn

Figure S11. Ex-situ SEM studies of the Sn0.8-ME5 optimized electrode: a,b) Higher magnification SEM micrographs in BSE mode for the pristine electrode (a) and the fully lithiated electrode (b) after 200 cycles. c,d) Secondary electrons (SE) SEM micrographs of the pristine electrode (c) and the fully lithiated electrode after 200 cycles (d), showing practically a lack of microcracks and no signs of a loss of connection with a current collector; Slight changes in a topography of the electrode's surface after cycling are ascribed to the contact with liquid electrolyte and DEC solvent, as well as to the fact there is lithium inserted into the material. e,f) BSE SEM micrographs together with the elemental distribution maps for the pristine electrode (e) and the fully lithiated electrode after 200 cycles (f), showing homogeneously distributed cations embedded in the carbon matrix for both electrodes before and after cycling.

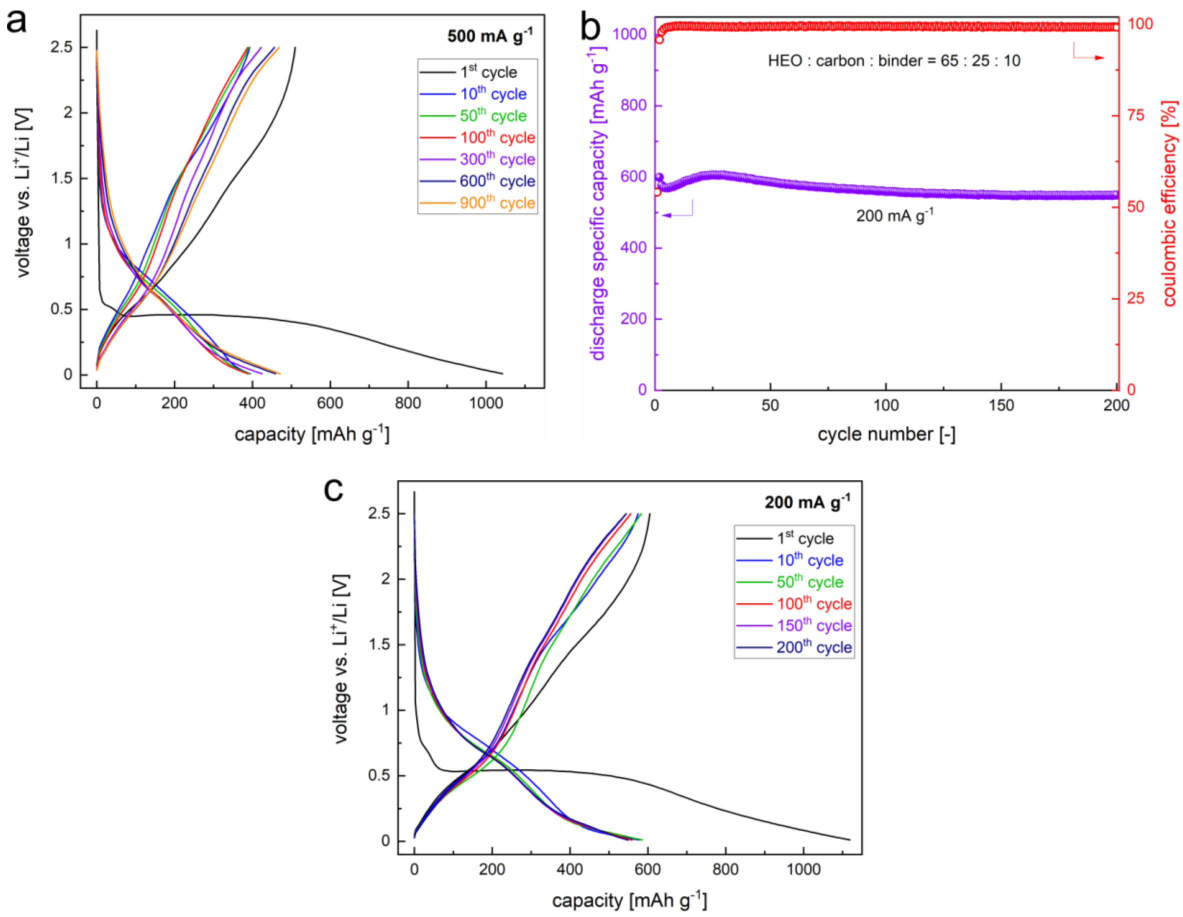

Figure S12. Cycling stability of the carbon content-optimized Sn0.8-ME5-based electrodes; the active material to carbon additive to binder ratio is 65:25:10, the binder is CMC/SBR and the electrolyte is 1M $LiPF_6$ in 1:1 (v/v) EC:DEC with 5 wt% FEC and 1 wt% VC addition. a) GDC curves corresponding to cycling presented in Figure 5f for 1st, 10th, 50th, 100th, 300th, 600th, and 900th cycles at 500 mA g^{-1}. b) Capacity retention under specific current of 200 mA g^{-1} the voltage range of 0.01-2.5 V for 200 cycles. c) Corresponding GDC curves for 1st, 10th, 50th, 100th, 150th, and 200th cycles at 200 mA g^{-1}.

Supplementary Note 10: Calculation of the theoretical capacity of Sn0.8-ME5 anode material

To calculate theoretical capacity of the 1st lithiation, it was assumed for the conversion reaction that all the elements from the initial spinel structure are reduced to the metallic state[4,5,57,84], except for the Mg^{2+}, which should be electrochemically inactive, forming MgO phase[13,36]. For the alloying reaction, it was assumed that all the Sn forms lithiated $Li_{4.4}Sn$ phase and all the Zn forms LiZn intermetallic[4,5,57,84].

To calculate the 1st lithiation capacity based on the observed changes of the oxidation state of the elements (established experimentally from XAS and Mössbauer measurements) it was assumed that ca. 70% of Sn remains at +4 oxidation state and 30% is reduced to the metallic state, and that the proportion between Mg^0 and Mg^{2+} is 1:1. We have not included the conventional alloying reaction in the calculations because we have not observed through ex-situ TEM and operando XRD studies formation of any crystalline intermetallic phases (e.g. Li_xSn_y, LiZn, Li_xMg_y), which are typical products of the alloying reaction. Because of the SEI layer formation and electrolyte decomposition (a well-known effect[22,85]), the exact value of lithiation level in the alloying process could not be established at this stage. Instead, the alloying and SEI formation capacity was estimated as a difference between the calculated capacity delivered through the conversion reaction and the experimental capacity value (taken as the value is the 1st discharge capacity for optimized electrode at the specific current of 50 mA g^{-1}, from the rate capability test, Figure 5e). It was found to be equal to ca. 492 mAh g^{-1}.

For the calculations of the reversible capacity based on the experimental results it was assumed that Mn and Zn are active elements in terms of the conversion reaction and reversibly change their oxidation states between +2 and 0. The difference in the reversible capacity calculated based on the occurring conversion reaction and the measured capacity (the value for 10th discharge capacity for optimized electrode at the specific current of 50 mA g^{-1} from the rate capability test, Figure 5e) is equal to 431 mAh g^{-1}, resulting in additional 4.4 mole of Li stored in the active material. Therefore, the conclusion is that this reversible storage occurs through an alloying-like reaction of the homogeneous, amorphous, and multicomponent matrix (that is without a change of the oxidation state of any particular element, but rather through electron exchange within the entire matrix).

- *Theoretical description of the 1st lithiation assuming conventional reactions:*

  <u>conversion</u>

  1) $Sn^{4+}_{0.8}(Co^{2+/3+}_{0.2}Mg^{2+}_{0.2}Mn^{2+/3+}_{0.2}Ni^{2+}_{0.2}Zn^{2+}_{0.2})_{2.2}O^{2-}_4$ + **7.12**Li^+ + 7.12e^- → $0.8Sn^0$ + $0.44Mg^{2+}O^{2-}$ + $3.56Li^+_2O^{2-}$ + $0.44Co^0$ + $0.44Mn^0$ + $0.44Ni^0$ + $0.44Zn^0$

alloying

2) $0.8Sn^{0}$ + **3.52**Li^{+} + $3.52e^{-}$ → $0.8Li_{4.4}Sn$

3) $0.44Zn^{0}$ + **0.44**Li^{+} + $0.44e^{-}$ → 0.44LiZn

total x = 11.08 (7.12+3.52+0.44)

$Q_{theoretical\_1st\_lithiation}$ = 1082 mAh g^{-1} (695 mAh g^{-1} from conversion and 387 mAh g^{-1} from alloying)

- *Description of the 1st lithiation:*

conversion

1) $Sn^{4+}_{0.8}(Co^{2+/3+}_{0.2}Mg^{2+}_{0.2}Mn^{2+/3+}_{0.2}Ni^{2+}_{0.2}Zn^{2+}_{0.2})_{2.2}O^{2-}_{4}$ + **5.32**Li^{+} + $5.32e^{-}$ → $0.56Sn^{4+}O^{2-}_{2}$ + $0.22Mg^{2+}O^{2-}$ + $2.66Li^{+}_{2}O^{2-}$ + $0.24Sn^{0}$ + $0.22Mg^{0}$ + $0.44Mn^{0}$ + $0.44Ni^{0}$ + $0.44Zn^{0}$

alloying

2) (amorphous matrix) + **x**Li^{+} + xe^{-} → Li_{x}(amorphous matrix)

$Q_{conversion\_1st\_lithiation}$ = 520 mAh g^{-1}

$Q_{experimental\_1st\_lithiation}$ = 1012 mAh g^{-1}

- *Description of the reversible lithiation:*

conversion

1) $0.44Mn^{2+}O^{2-}$ + **0.88**Li^{+} + $0.88e^{-}$ ↔ Mn^{0} + $0.44Li^{+}_{2}O^{2-}$

2) $0.44Zn^{2+}O^{2-}$ + **0.88**Li^{+} + $0.88e^{-}$ ↔ Zn^{0} + $0.44Li^{+}_{2}O^{2-}$

alloying

3) (amorphous matrix) + **4.4**Li^{+} + $4.4e^{-}$ → $Li_{4.4}$(amorphous matrix)

$Q_{conversion\_reversible}$ = 176 mAh g^{-1}

$Q_{reversible\_experimental}$ = 603 mAh g^{-1}

References

(1) Baggetto, L.; Niessen, R. A. H.; Roozehoom, F.; Notten, P. H. L. High Energy Density All-Solid-State Batteries: A Challenging Concept towards 3D Integration. *Adv. Funct. Mater.* **2008**, *18* (7), 1057–1066. https://doi.org/10.1002/adfm.200701245.

(2) Wang, J.; King, P.; Huggins, R. A. Investigations of Binary Lithium-Zinc, Lithium-Cadmium and Lithium-Lead Alloys as Negative Electrodes in Organic Solvent-Based Electrolyte. *Solid State Ionics* **1986**, *20* (3), 185–189. https://doi.org/10.1016/0167-2738(86)90212-2.

(3) Wang, J.; Raistrick, I. D.; Huggins, R. A. Behavior of Some Binary Lithium Alloys As Negative Electrodes in Organic Solvent Based Electrolytes. *Electrochem. Soc. Ext. Abstr.* **1984**, *84–2*, 184–185. https://doi.org/10.1149/1.2108601.

(4) Bresser, D.; Passerini, S.; Scrosati, B. Leveraging Valuable Synergies by Combining Alloying and Conversion for Lithium-Ion Anodes. *Energy Environ. Sci.* **2016**, *9* (11), 3348–3367. https://doi.org/10.1039/c6ee02346k.

(5) Reddy, M. V.; Subba Rao, G. V.; Chowdari, B. V. R. Metal Oxides and Oxysalts as Anode Materials for Li Ion Batteries. *Chem. Rev.* **2013**, *113* (7), 5364–5457. https://doi.org/10.1021/cr3001884.

(6) Sun, Y.; Liu, N.; Cui, Y. Promises and Challenges of Nanomaterials for Lithium-Based Rechargeable Batteries. *Nat. Energy* **2016**, *1* (7). https://doi.org/10.1038/nenergy.2016.71.

(7) Obrovac, M. N.; Chevrier, V. L. Alloy Negative Electrodes for Li-Ion Batteries. *Chem. Rev.* **2014**, *114* (23), 11444–11502. https://doi.org/10.1021/cr500207g.

(8) Asenbauer, J.; Eisenmann, T.; Kuenzel, M.; Kazzazi, A.; Chen, Z.; Bresser, D. The Success Story of Graphite as a Lithium-Ion Anode Material – Fundamentals, Remaining Challenges, and Recent Developments Including Silicon (Oxide) Composites. *Sustain. Energy Fuels* **2020**, *4* (11), 5387–5416. https://doi.org/10.1039/d0se00175a.

(9) Bresser, D.; Paillard, E.; Niehoff, P.; Krueger, S.; Mueller, F.; Winter, M.; Passerini, S. Challenges of "Going Nano": Enhanced Electrochemical Performance of Cobalt Oxide Nanoparticles by Carbothermal Reduction and in Situ Carbon Coating. *ChemPhysChem* **2014**, *15* (10), 2177–2185. https://doi.org/10.1002/cphc.201400092.

(10) Zhuang, Y.; Ma, Z.; Deng, Y.; Song, X.; Zuo, X.; Xiao, X.; Nan, J. Sandwich-like Mn_3O_4/Carbon Nanofragment Composites with a Higher Capacity than Commercial Graphite and Hierarchical Voltage Plateaus for Lithium Ion Batteries. *Electrochim. Acta* **2017**, *245*, 448–455. https://doi.org/10.1016/j.electacta.2017.05.171.

(11) Song, Y.; Hwang, J.; Lee, S.; Thirumalraj, B.; Kim, J. H.; Jenei, P.; Gubicza, J.; Choe, H. Synthesis of a High-Capacity NiO/Ni Foam Anode for Advanced Lithium-Ion Batteries. *Adv. Eng. Mater.* **2020**, *22* (11), 1–8. https://doi.org/10.1002/adem.202000351.

(12) Cao, K.; Jin, T.; Yang, L.; Jiao, L. Recent Progress in Conversion Reaction Metal Oxide Anodes for Li-Ion Batteries. *Mater. Chem. Front.* **2017**, *1* (11), 2213–2242. https://doi.org/10.1039/c7qm00175d.

(13) Sarkar, A.; Velasco, L.; Wang, D.; Wang, Q.; Talasila, G.; de Biasi, L.; Kübel, C.; Brezesinski, T.; Bhattacharya, S. S.; Hahn, H.; Breitung, B. High Entropy Oxides for Reversible Energy Storage. *Nat. Commun.* **2018**, *9* (1). https://doi.org/10.1038/s41467-018-05774-5.

(14) Wang, D.; Jiang, S.; Duan, C.; Mao, J.; Dong, Y.; Dong, K.; Wang, Z.; Luo, S.; Liu, Y.; Qi, X. Spinel-Structured High Entropy Oxide $(FeCoNiCrMn)_3O_4$ as Anode towards Superior Lithium Storage Performance. *J. Alloys Compd.* **2020**, *844*, 156158. https://doi.org/10.1016/j.jallcom.2020.156158.

(15) Qiu, N.; Chen, H.; Yang, Z.; Sun, S.; Wang, Y.; Cui, Y. A High Entropy Oxide $(Mg_{0.2}Co_{0.2}Ni_{0.2}Cu_{0.2}Zn_{0.2}O)$ with Superior Lithium Storage Performance. *J. Alloys Compd.* **2019**, *777*, 767–774. https://doi.org/10.1016/j.jallcom.2018.11.049.

(16) Wang, S.-Y.; Chen, T.-Y.; Kuo, C.-H.; Lin, C.-C.; Huang, S.-C.; Lin, M.-H.; Wang, C.-C.; Chen, H.-Y. Operando Synchrotron Transmission X-Ray Microscopy Study on (Mg, Co, Ni, Cu, Zn)O High-Entropy Oxide Anodes for Lithium-Ion Batteries. *Mater. Chem. Phys.* **2021**, 125105. https://doi.org/10.1016/j.matchemphys.2021.125105.

(17) Ghigna, P.; Airoldi, L.; Fracchia, M.; Callegari, D.; Anselmi-Tamburini, U.; D'angelo, P.; Pianta, N.; Ruffo, R.; Cibin, G.; De Souza, D. O.; Quartarone, E. Lithiation Mechanism in High-Entropy Oxides as Anode Materials for Li-Ion Batteries: An Operando XAS Study. *ACS Appl. Mater. Interfaces* **2020**, *12* (45), 50344–50354. https://doi.org/10.1021/acsami.0c13161.

(18) Duan, C. Q.; Tian, K.; Li, X.; Wang, D.; Sun, H.; Zheng, R.; Wang, Z.; Liu, Y. New Spinel High-Entropy Oxides $(FeCoNiCrMn_XLi)_3O_4$ (X = Cu, Mg, Zn) as the Anode Material for Lithium-Ion Batteries. *Ceram. Int.* **2021**, *47* (22), 32025–32032. https://doi.org/10.1016/j.ceramint.2021.08.091.

(19) Xiang, H.-Z.; Xie, H.-X.; Chen, Y.-X.; Zhang, H.; Mao, A.; Zheng, C.-H. Porous Spinel-Type $(Al_{0.2}CoCrFeMnNi)_{0.58}O_{4-\delta}$ High-Entropy Oxide as a Novel High-Performance Anode Material for Lithium-Ion Batteries. *J. Mater. Sci.* **2021**, *56* (13), 8127–8142. https://doi.org/10.1007/s10853-021-05805-5.

(20) Chen, H.; Qiu, N.; Wu, B.; Yang, Z.; Sun, S.; Wang, Y. A New Spinel High-Entropy Oxide $(Mg_{0.2}Ti_{0.2}Zn_{0.2}Cu_{0.2}Fe_{0.2})_3O_4$ with Fast Reaction Kinetics and Excellent Stability as an Anode Material for Lithium Ion Batteries. *RSC Adv.* **2020**, *10* (16), 9736–9744. https://doi.org/10.1039/d0ra00255k.

(21) Chen, T.-Y.; Wang, S.-Y.; Kuo, C.-H.; Huang, S.-C.; Lin, M.-H.; Li, C.-H.; Chen, H.-Y. T.; Wang, C.-C.; Liao, Y.-F.; Lin, C.-C.; Chang, Y.-M.; Yeh, J.-W.; Lin, S.-J.; Chen, T.-Y.; Chen, H.-Y. In Operando Synchrotron X-Ray Studies of a Novel Spinel $(Ni_{0.2}Co_{0.2}Mn_{0.2}Fe_{0.2}Ti_{0.2})_3O_4$ High-Entropy Oxide for Energy Storage Applications . *J. Mater. Chem. A* **2020**, *8* (41), 21756–21770. https://doi.org/10.1039/d0ta06455f.

(22) Nguyen, T. X.; Patra, J.; Chang, J. K.; Ting, J. M. High Entropy Spinel Oxide Nanoparticles for Superior Lithiation-Delithiation Performance. *J. Mater. Chem. A* **2020**, *8* (36), 18963–18973. https://doi.org/10.1039/d0ta04844e.

(23) Huang, C. Y.; Huang, C. W.; Wu, M. C.; Patra, J.; Xuyen Nguyen, T.; Chang, M. T.;

Clemens, O.; Ting, J. M.; Li, J.; Chang, J. K.; Wu, W. W. Atomic-Scale Investigation of Lithiation/Delithiation Mechanism in High-Entropy Spinel Oxide with Superior Electrochemical Performance. *Chem. Eng. J.* **2021**, *420* (February). https://doi.org/10.1016/j.cej.2021.129838.

(24) Moździerz, M.; Dąbrowa, J.; Stępień, A.; Zajusz, M.; Stygar, M.; Zając, W.; Danielewski, M.; Świerczek, K. Mixed Ionic-Electronic Transport in the High-Entropy $(Co,Cu,Mg,Ni,Zn)_{1-x}Li_xO$ Oxides. *Acta Mater.* **2021**, *208*, 116735. https://doi.org/10.1016/j.actamat.2021.116735.

(25) Wang, B. Y.; Wang, H. Y.; Ma, Y. L.; Zhao, X. H.; Qi, W.; Jiang, Q. C. Facile Synthesis of Fine Zn_2SnO_4 Nanoparticles/Graphene Composites with Superior Lithium Storage Performance. *J. Power Sources* **2015**, *281*, 341–349. https://doi.org/10.1016/j.jpowsour.2015.02.014.

(26) Cherian, C. T.; Zheng, M.; Reddy, M. V.; Chowdari, B. V. R.; Sow, C. H. Zn_2SnO_4 Nanowires versus Nanoplates: Electrochemical Performance and Morphological Evolution during Li-Cycling. *ACS Appl. Mater. Interfaces* **2013**, *5* (13), 6054–6060. https://doi.org/10.1021/am400802j.

(27) Zhang, J.; Liang, J.; Zhu, Y.; Wei, D.; Fan, L.; Qian, Y. Synthesis of Co_2SnO_4 Hollow Cubes Encapsulated in Graphene as High Capacity Anode Materials for Lithium-Ion Batteries. *J. Mater. Chem. A* **2014**, *2* (8), 2728–2734. https://doi.org/10.1039/c3ta13228e.

(28) Chen, C.; Ru, Q.; Hu, S.; An, B.; Song, X.; Hou, X. Co_2SnO_4 Nanocrystals Anchored on Graphene Sheets as High-Performance Electrodes for Lithium-Ion Batteries. *Electrochim. Acta* **2015**, *151*, 203–213. https://doi.org/10.1016/j.electacta.2014.11.018.

(29) Xiao, T.; Tang, Y.; Jia, Z.; Feng, S. Synthesis of SnO_2/Mg_2SnO_4 Nanoparticles and Their Electrochemical Performance for Use in Li-Ion Battery Electrodes. *Electrochim. Acta* **2009**, *54* (8), 2396–2401. https://doi.org/10.1016/j.electacta.2008.10.061.

(30) Tang, H.; Cheng, C.; Yu, G.; Liu, H.; Chen, W. Structure and Electrochemical Properties of Mg_2SnO_4 Nanoparticles Synthesized by a Facile Co-Precipitation Method. *Mater. Chem. Phys.* **2015**, *159*, 167–172. https://doi.org/10.1016/j.matchemphys.2015.03.066.

(31) Lei, S.; Tang, K.; Chen, C.; Jin, Y.; Zhou, L. Preparation of Mn_2SnO_4 Nanoparticles as the Anode Material for Lithium Secondary Battery. *Mater. Res. Bull.* **2009**, *44* (2), 393–397. https://doi.org/10.1016/j.materresbull.2008.05.006.

(32) Hua, C.; Fang, X.; Wang, Z.; Chen, L. Transition-Metal-Catalyzed Oxidation of Metallic Sn in NiO/SnO_2 Nanocomposite. *Chem. - A Eur. J.* **2014**, *20* (18), 5487–5491. https://doi.org/10.1002/chem.201304817.

(33) Šepelák, V.; Becker, S. M.; Bergmann, I.; Indris, S.; Scheuermann, M.; Feldhoff, A.; Kübel, C.; Bruns, M.; Stürzl, N.; Ulrich, A. S.; Ghafari, M.; Hahn, H.; Grey, C. P.; Becker, K. D.; Heitjans, P. Nonequilibrium Structure of Zn_2SnO_4 Spinel Nanoparticles. *J. Mater. Chem.* **2012**, *22* (7), 3117–3126. https://doi.org/10.1039/c2jm15427g.

(34) Zhang, W. J. A Review of the Electrochemical Performance of Alloy Anodes for Lithium-Ion Batteries. *J. Power Sources* **2011**, *196* (1), 13–24.

https://doi.org/10.1016/j.jpowsour.2010.07.020.

(35) Zhang, Z.; Zhao, M.; Xia, M.; Qi, R.; Liu, M.; Nie, J.; Wang, Z. L.; Lu, X. Magnesium Anodes with Extended Cycling Stability for Lithium-Ion Batteries. *Adv. Funct. Mater.* **2019**, *29* (41), 1–9. https://doi.org/10.1002/adfm.201806400.

(36) Zhou, W.; Upreti, S.; Whittingham, M. S. High Performance Si/MgO/Graphite Composite as the Anode for Lithium-Ion Batteries. *Electrochem. commun.* **2011**, *13* (10), 1102–1104. https://doi.org/10.1016/j.elecom.2011.07.006.

(37) Becker, S. M.; Scheuermann, M.; Sepelák, V.; Eichhöfer, A.; Chen, D.; Mönig, R.; Ulrich, A. S.; Hahn, H.; Indris, S. Electrochemical Insertion of Lithium in Mechanochemically Synthesized Zn_2SnO_4. *Phys. Chem. Chem. Phys.* **2011**, *13* (43), 19624–19631. https://doi.org/10.1039/c1cp22298h.

(38) Huang, Z.; Gao, H.; Yang, Z.; Jiang, W.; Wang, Q.; Wang, S.; Ju, J.; Kwon, Y. U.; Zhao, Y. Improved Capacity and Cycling Stability of SnO_2 Nanoanode Induced by Amorphization during Cycling for Lithium Ion Batteries. *Mater. Des.* **2019**, *180*, 107973. https://doi.org/10.1016/j.matdes.2019.107973.

(39) Kim, C.; Noh, M.; Choi, M.; Cho, J.; Park, B. Critical Size of a Nano SnO_2 Electrode for Li-Secondary Battery. *Chem. Mater.* **2005**, *17* (12), 3297–3301. https://doi.org/10.1021/cm048003o.

(40) Lou, X. W.; Wang, Y.; Yuan, C.; Lee, J. Y.; Archer, L. A. Template-Free Synthesis of SnO_2 Hollow Nanostructures with High Lithium Storage Capacity. *Adv. Mater.* **2006**, *18* (17), 2325–2329. https://doi.org/10.1002/adma.200600733.

(41) Shen, X.; Shen, J.; You, S. J.; Yang, L. X.; Tang, L. Y.; Li, Y. C.; Liu, J.; Yang, H.; Zhu, K.; Liu, Y. L.; Zhou, W. Y.; Yu, R. C.; Xie, S. S.; Shen, X.; Shen, J.; You, S. J.; Yang, L. X.; Tang, L. Y.; Li, Y. C.; Liu, J.; Yang, H. Phase Transition of Zn_2SnO_4 Nanowires under High Pressure. **2014**, *113523* (2009). https://doi.org/10.1063/1.3268460.

(42) Ma, Q.; Wu, S.; Fan, Y. Synthesis and Microwave Dielectric Properties of Zn_2SnO_4 Ceramics. *Ceram. Int.* **2014**, *40* (1 PART A), 1073–1080. https://doi.org/10.1016/j.ceramint.2013.06.106.

(43) D'Ippolito, V.; Andreozzi, G. B.; Bersani, D.; Lottici, P. P. Raman Fingerprint of Chromate, Aluminate and Ferrite Spinels. *J. Raman Spectrosc.* **2015**, *46* (12), 1255–1264. https://doi.org/10.1002/jrs.4764.

(44) Dąbrowa, J.; Stygar, M.; Mikuła, A.; Knapik, A.; Mroczka, K.; Tejchman, W.; Danielewski, M.; Martin, M. Synthesis and Microstructure of the $(Co,Cr,Fe,Mn,Ni)_3O_4$ high Entropy Oxide Characterized by Spinel Structure. *Mater. Lett.* **2018**, *216*, 32–36. https://doi.org/10.1016/j.matlet.2017.12.148.

(45) Madern, N.; Monnier, J.; Baddour-Hadjean, R.; Steckmeyer, A.; Joubert, J. M. Characterization of Refractory Steel Oxidation at High Temperature. *Corros. Sci.* **2018**, *132* (March), 223–233. https://doi.org/10.1016/j.corsci.2017.12.029.

(46) Laguna-Bercero, M. A.; Sanjuan, M. L.; Merino, R. I. Raman Spectroscopic Study of Cation Disorder in Poly- and Single Crystals of the Nickel Aluminate Spinel. *J. Phys.: Condens.*

Matter 2007, 186217. https://doi.org/10.1088/0953-8984/19/18/186217.

(47) Usharani, N. J.; Sanghavi, H.; Bhattacharya, S. S. Factors Influencing Phase Formation and Band Gap Studies of a Novel Multicomponent High Entropy (Co,Cu,Mg,Ni,Zn)$_2$TiO$_4$ Orthotitanate Spinel. *J. Alloys Compd.* **2021**, *888*, 161390. https://doi.org/10.1016/j.jallcom.2021.161390.

(48) Lazzeri, M.; Thibaudeau, P. Ab Initio Raman Spectrum of the Normal and Disordered MgAl$_2$O$_4$ Spinel. *Phys. Rev. B - Condens. Matter Mater. Phys.* **2006**, *74* (14), 2–5. https://doi.org/10.1103/PhysRevB.74.140301.

(49) Hosterman, B. D. Raman Spectroscopic Study of Solid Solution Spinel Oxides, UNLV Theses, Dissertations, Professional Papers, and Capstones. **2011**.

(50) Rost, C. M.; Rak, Z.; Brenner, D. W.; Maria, J. P. Local Structure of the Mg$_x$Ni$_x$Co$_x$Cu$_x$Zn$_x$O(X=0.2) Entropy-Stabilized Oxide: An EXAFS Study. *J. Am. Ceram. Soc.* **2017**, *100* (6), 2732–2738. https://doi.org/10.1111/jace.14756.

(51) Berardan, D.; Meena, A. K.; Franger, S.; Herrero, C.; Dragoe, N. Controlled Jahn-Teller Distortion in (MgCoNiCuZn)O-Based High Entropy Oxides. *J. Alloys Compd.* **2017**, *704*, 693–700. https://doi.org/10.1016/j.jallcom.2017.02.070.

(52) Quinn, E. C. O.; Shamblin, J.; Perlov, B.; Ewing, R. C.; Neuefeind, J.; Feygenson, M.; Gussev, I.; Lang, M. Inversion in Mg 1 −. **2017**, 1–8. https://doi.org/10.1021/jacs.7b04370.

(53) Ivanov, V. G.; Abrashev, M. V; Iliev, M. N.; Gospodinov, M. M.; Meen, J.; Aroyo, M. I. Short-Range B -Site Ordering in the Inverse Spinel Ferrite NiFe 2 O 4. **2010**, 1–8. https://doi.org/10.1103/PhysRevB.82.024104.

(54) Fu, M.; Ma, X.; Zhao, K.; Li, X.; Su, D. High-Entropy Materials for Energy-Related Applications. *iScience* **2021**, *24* (3), 102177. https://doi.org/10.1016/j.isci.2021.102177.

(55) Dąbrowa, J.; Cieślak, J.; Zajusz, M.; Moździerz, M.; Berent, K.; Mikuła, A.; Stępień, A.; Świerczek, K. Structure and Transport Properties of the Novel (Dy,Er,Gd,Ho,Y)$_3$Fe$_5$O$_{12}$ and (Dy,Gd,Ho,Sm,Y)$_3$Fe$_5$O$_{12}$ High Entropy Garnets. *J. Eur. Ceram. Soc.* **2021**, *41* (6), 3844–3849. https://doi.org/10.1016/j.jeurceramsoc.2020.12.052.

(56) Cieslak, J.; Reissner, M.; Berent, K.; Dabrowa, J.; Stygar, M.; Mozdzierz, M.; Zajusz, M. Magnetic Properties and Ionic Distribution in High Entropy Spinels Studied by Mössbauer and Ab Initio Methods. *Acta Mater.* **2021**, *206*, 116600. https://doi.org/10.1016/j.actamat.2020.116600.

(57) Alcántara, R.; Ortiz, G. F.; Lavela, P.; Tirado, J. L. Electrochemical and 119Sn Mössbauer Studies of the Reaction of Co$_2$SnO$_4$ with Lithium. *Electrochem. commun.* **2006**, *8* (5), 731–736. https://doi.org/10.1016/j.elecom.2006.02.024.

(58) Lavela, P.; Pérez-Vicente, G.; Tirado, J. L.; Branci, C.; Olivier-Fourcade, J.; Jumas, J. C. Structural Characterization and Electrochemical Reactions with Lithium of Cu$_2$CoTi$_x$Sn$_{3-x}$S$_8$ Solid Solutions. *Chem. Mater.* **1999**, *11* (10), 2687–2693. https://doi.org/10.1021/cm990003e.

(59) Young, D. L.; Williamson, D. L.; Coutts, T. J. Structural Characterization of Zinc Stannate

Thin Films. *J. Appl. Phys.* **2002**, *91* (3), 1464–1471. https://doi.org/10.1063/1.1429793.

(60) Chouvin, J.; Olivier-Fourcade, J.; Jumas, J. C.; Simon, B.; Biensan, P.; Fernández Madrigal, F. J.; Tirado, J. L.; Pérez Vicente, C. SnO Reduction in Lithium Cells: Study by X-Ray Absorption, 119Sn Mössbauer Spectroscopy and X-Ray Diffraction. *J. Electroanal. Chem.* **2000**, *494* (2), 136–146. https://doi.org/10.1016/S0022-0728(00)00357-0.

(61) Chouvin, J.; Olivier-Fourcade, J.; Jumas, J. .; Simon, B.; Godiveau, O. 119Sn Mössbauer Study of Li_xSn Alloys Prepared Electrochemically. *Chem. Phys. Lett.* **1999**, *308* (5–6), 413–420. https://doi.org/10.1016/s0009-2614(99)00632-6.

(62) Sepelak, V.; Becker, S. M.; Bergmann, I.; Indris, S.; Scheuermann, M.; Feldhoff, A.; Christian, K.; Bruns, M.; St, N.; Ulrich, A. S.; Ghafari, M.; Hahn, H.; Grey, C. P.; Becker, D.; Heitjans, P. Nonequilibrium structure of Zn_2SnO_4 spinel nanoparticles. *Journal of Materials Chemistry*. **2012**, 3117–3126. https://doi.org/10.1039/c2jm15427g.

(63) Jie, L., Chao, X., XPS Examination of Tin Oxide on Float Glass Surface, 119 (1990) 37-40. *Jie, L., Chao, X., XPS Exam. tin oxide float Glas. surface, 119 37-40* **1990**, *119*, 37–40.

(64) Wagner, A.D., Naumkin, A.V., Kraut-Vass, A., Allison, J.W., Powell, C.J., Rumble, J.R.J., NIST Standard Reference Database 20, in, Http:/Srdata.Nist.Gov/Xps/, 2003.

(65) Whittles, T. J.; Burton, L. A.; Skelton, J. M.; Walsh, A.; Veal, T. D.; Dhanak, V. R. Band Alignments, Valence Bands, and Core Levels in the Tin Sulfides SnS, SnS_2, and Sn_2S_3: Experiment and Theory. *Chem. Mater.* **2016**, *28* (11), 3718–3726. https://doi.org/10.1021/acs.chemmater.6b00397.

(66) Biesinger, M. C.; Lau, L. W. M.; Gerson, A. R.; Smart, R. S. C. Resolving Surface Chemical States in XPS Analysis of First Row Transition Metals, Oxides and Hydroxides: Sc, Ti, V, Cu and Zn. *Appl. Surf. Sci.* **2010**, *257* (3), 887–898. https://doi.org/10.1016/j.apsusc.2010.07.086.

(67) Biesinger, M. C.; Payne, B. P.; Grosvenor, A. P.; Lau, L. W. M.; Gerson, A. R.; Smart, R. S. C. Resolving Surface Chemical States in XPS Analysis of First Row Transition Metals, Oxides and Hydroxides: Cr, Mn, Fe, Co and Ni. *Appl. Surf. Sci.* **2011**, *257* (7), 2717–2730. https://doi.org/10.1016/j.apsusc.2010.10.051.

(68) Okamoto, Y., Adachi, T., Maezawa, A., Imanaka, T., Effect of ZnO Addition on Cobalt–Alumina Interaction Species, Bulletin of the Chemical Society of Japan, 64 (1991) 236-242.

(69) Dash, K.C., Folkesson, B., Larsson, R., Mohapatra, M. An XPS Investigation on a Series of Schiff Base Dioxime Ligands and Cobalt Complexes. *Inorg. Chem.* **1989**, *49*, 343–357.

(70) Biesinger, M. C.; Payne, B. P.; Lau, L. W. M.; Gerson, A.; Smart, R. S. C. X-Ray Photoelectron Spectroscopic Chemical State Quantification of Mixed Nickel Metal, Oxide and Hydroxide Systems. *Surf. Interface Anal.* **2009**, *41* (4), 324–332. https://doi.org/10.1002/sia.3026.

(71) Chen, X.; Li, L.; Liu, M.; Huang, T.; Yu, A. Detection of Lithium Plating in Lithium-Ion Batteries by Distribution of Relaxation Times. *J. Power Sources* **2021**, *496* (March), 229867. https://doi.org/10.1016/j.jpowsour.2021.229867.

(72) Pan, K.; Zou, F.; Canova, M.; Zhu, Y.; Kim, J. H. Comprehensive Electrochemical Impedance Spectroscopy Study of Si-Based Anodes Using Distribution of Relaxation Times Analysis. *J. Power Sources* **2020**, *479* (October), 229083. https://doi.org/10.1016/j.jpowsour.2020.229083.

(73) Kaisar, N.; Paul, T.; Chi, P.; Su, Y.; Singh, A.; Chu, C.; Wu, M.; Wu, P. M. Perovskite in Li-Ion Batteries. **2021**.

(74) Schönleber, M.; Klotz, D.; Ivers-Tiffée, E. A Method for Improving the Robustness of Linear Kramers-Kronig Validity Tests. *Electrochim. Acta* **2014**, *131*, 20–27. https://doi.org/10.1016/j.electacta.2014.01.034.

(75) Courtney, I. A.; Dunlap, R. A.; Dahn, J. R. In-Situ 119Sn Mössbauer Effect Studies of the Reaction of Lithium with SnO and SnO:0.25 B_2O_3:0.25 P_2O_5 Glass. *Electrochim. Acta* **1999**, *45* (1–2), 51–58. https://doi.org/10.1016/S0013-4686(99)00192-9.

(76) Dunlap, R. A.; Small, D. A.; MacNeil, D. D.; Obrovac, M. N.; Dahn, J. R. Moessbauer Effect Investigation of the Li-Sn System. *J. Alloys Compd.* **1999**, *289* (1–2), 135–142. https://doi.org/10.1016/S0925-8388(99)00165-6.

(77) Kang, J. S.; Kim, G.; Wi, S. C.; Lee, S. S.; Choi, S.; Cho, S.; Han, S. W.; Kim, K. H.; Song, H. J.; Shin, H. J.; Sekiyama, A.; Kasai, S.; Suga, S.; Min, B. I. Spatial Chemical Inhomogeneity and Local Electronic Structure of Mn-Doped Ge Ferromagnetic Semiconductors. *Phys. Rev. Lett.* **2005**, *94* (14), 3–6. https://doi.org/10.1103/PhysRevLett.94.147202.

(78) Gilbert, B.; Frazer, B. H.; Belz, A.; Conrad, P. G.; Nealson, K. H.; Haskel, D.; Lang, J. C.; Srajer, G.; De Stasio, G. Multiple Scattering Calculations of Bonding and X-Ray Absorption Spectroscopy of Manganese Oxides. *J. Phys. Chem. A* **2003**, *107* (16), 2839–2847. https://doi.org/10.1021/jp021493s.

(79) Sánchez-Agudo, M.; Yubero, F.; Fuentes, G. G.; Gutiérrez, A.; Sacchi, M.; Soriano, L.; Sanz, J. M. Study of the Growth of Ultrathin Films of NiO on Cu(111). *Surf. Interface Anal.* **2000**, *30* (1), 396–400. https://doi.org/10.1002/1096-9918(200008)30:1<396::AID-SIA804>3.0.CO;2-3.

(80) Eisenmann, T.; Asenbauer, J.; Rezvani, S. J.; Diemant, T.; Behm, R. J.; Geiger, D.; Kaiser, U.; Passerini, S.; Bresser, D. Impact of the Transition Metal Dopant in Zinc Oxide Lithium-Ion Anodes on the Solid Electrolyte Interphase Formation. *Small Methods* **2021**, *5* (4), 21–25. https://doi.org/10.1002/smtd.202001021.

(81) Chiou, J. W.; Tsai, H. M.; Pao, C. W.; Krishna Kumar, K. P.; Ray, S. C.; Chien, F. Z.; Pong, W. F.; Tsai, M. H.; Chen, C. H.; Lin, H. J.; Wu, J. J.; Yang, M. H.; Liu, S. C.; Chiang, H. H.; Chen, C. W. Comparison of the Electronic Structures of $Zn_{1-x}Co_xO$ and $Zn_{1-x}Mg_xO$ Nanorods Using X-Ray Absorption and Scanning Photoelectron Microscopies. *Appl. Phys. Lett.* **2006**, *89* (4), 2–5. https://doi.org/10.1063/1.2240108.

(82) Liu, X. C.; Shi, E. W.; Chen, Z. Z.; Chen, B. Y.; Huang, W.; Song, L. X.; Zhou, K. J.; Cui, M. Q.; Xie, Z.; He, B.; Wei, S. Q. The Local Structure of Co-Doped ZnO Films Studied by X-Ray Absorption Spectroscopy. *J. Alloys Compd.* **2008**, *463* (1–2), 435–439. https://doi.org/10.1016/j.jallcom.2007.09.030.

(83) Qiao, R.; Wray, L. A.; Kim, J. H.; Pieczonka, N. P. W.; Harris, S. J.; Yang, W. Direct Experimental Probe of the Ni(II)/Ni(III)/Ni(IV) Redox Evolution in $LiNi_{0.5}Mn_{1.5}O_4$ Electrodes. *J. Phys. Chem. C* **2015**, *119* (49), 27228–27233. https://doi.org/10.1021/acs.jpcc.5b07479.

(84) Huang, J. Y.; Zhong, L.; Wang, C. M.; Sullivan, J. P.; Xu, W.; Zhang, L. Q.; Mao, S. X.; Hudak, N. S.; Liu, X. H.; Subramanian, A.; Fan, H.; Qi, L.; Kushima, A.; Li, J. In Situ Observation of the Electrochemical Lithiation of a Single SnO_2 Nanowire Electrode. *Science (80-.).* **2010**, *330* (6010), 1515–1520. https://doi.org/10.1126/science.1195628.

(85) Chen, H.; Qiu, N.; Wu, B.; Yang, Z.; Sun, S.; Wang, Y. Tunable Pseudocapacitive Contribution by Dimension Control in Nanocrystalline-Constructed $(Mg_{0.2}Co_{0.2}Ni_{0.2}Cu_{0.2}Zn_{0.2})O$ Solid Solutions to Achieve Superior Lithium-Storage Properties. *RSC Adv.* **2019**, *9* (50), 28908–28915. https://doi.org/10.1039/c9ra05508h.
